# Local adaptation, genetic diversity and key environmental interactions in a collection of novel red clover germplasm

**DOI:** 10.3389/fpls.2025.1553094

**Published:** 2025-03-17

**Authors:** A. D. Heslop, Sai K. Arojju, Rainer W. Hofmann, John L. Ford, M. Zulfi Z. Jahufer, Anna C. Larking, Rachael Ashby, Charles A. Hefer, Ken G. Dodds, A. Saei, Jessica O’Connor, Andrew G. Griffiths

**Affiliations:** ^1^ Faculty of Agriculture and Life Sciences, Lincoln University, Lincoln, New Zealand; ^2^ AgResearch Limited, Lincoln Research Centre, Christchurch, New Zealand; ^3^ Radiata Pine Breeding Company, Building EN27, University of Canterbury, Christchurch, New Zealand; ^4^ PGG Wrightson Seeds Limited, C/- Grasslands Research Centre, Palmerston North, New Zealand; ^5^ School of Agriculture and Food Sustainability, The University of Queensland, Brisbane, QLD, Australia; ^6^ AgResearch Ltd., Grasslands Research Centre, Palmerston North, New Zealand; ^7^ AgResearch Limited, Invermay Agricultural Centre, Mosgiel, New Zealand

**Keywords:** red clover (*Trifolium pratense* L.), genetic diversity, heritability, genotype by-sequencing, germplasm, landscape genomics, redundancy analysis

## Abstract

Red clover (*Trifolium pratense* L.) is known for its large taproot, nitrogen fixation capabilities and production of forage high in protein and digestibility. It has the potential to strengthen temperate pastural systems against future adverse climatic events by providing higher biomass during periods of water deficit. Being outcrossing and self-incompatible, red clover is a highly heterozygous species. If evaluated and utilized correctly, this genetic diversity can be harnessed to develop productive, persistent cultivars. In this study, we selected 92 geographically diverse red clover novel germplasm populations for assessment in multi-location, multi-year field trials and for genetic diversity and genetic relationship characterization using pooled genotyping-by-sequencing (GBS). Through the development of integrated linear mixed models based on genomic, phenotypic, and environmental information we assessed variance components and genotype-by-environment (G x E) interactions for eight physiological and morphological traits. Key interactions between environmental variables and plant performance were also evaluated using a common garden site at Lincoln. We found that the genetic structure of the 92 populations was highly influenced by country of origin. The expected heterozygosity within populations ranged between 0.08 and 0.17 and varied with geographical origin. For the eight physiological and morphological traits measured there was high narrow-sense heritability (*h^2^* > 0.70). The influence of environmental variables, such as mean precipitation, temperature and isothermality of the original collection locations, on plant and trait performance in the local field trials was also highlighted. Along with the identification of genes associated with these bioclimatic variables that could be used as genetic markers for selection in future breeding programs. Our study identifies the importance of diverse germplasm when adding genetic variation into breeding programs. It also identifies efficient evaluation methods and key climatic variables that should be considered when developing adaptive red clover cultivars.

## Introduction

Red clover (*Trifolium pratense* L.) is an important forage legume species used globally in both pure and mixed stand pasture systems (Taylor and Quesenberry, 1996). As a legume, red clover can fix atmospheric nitrogen biologically to generate plant-available nitrogen through symbiosis with soil-dwelling *Rhizobium* bacteria (Taylor and Quesenberry, 1996). This plant species is high in protein, readily digestible, and can grow in a wide range of soil types, pH levels and environmental conditions (Taylor and Quesenberry, 1996). It is thought to have originated from the Eastern Mediterranean region and spread throughout Europe and western Asia and is now established throughout most temperate regions in the world (Annicchiarico et al., 2015; Taylor and Quesenberry, 1996). Earliest reports of red clover cultivation date to the 16^th^ century in Spain, where farmers collected and spread naturalized red clover for the improvement of pasture stands or for adding into the crop rotation. These landraces possess wide variation for adaptive and agronomic traits (Annicchiarico et al., 2015; Taylor and Quesenberry, 1996). Adaptive landraces collected thus far have either been integrated effectively into breeding programs or are currently conserved in genebanks worldwide (Nay et al., 2023). Similar to breeding programs of other economically important species, further exploration of novel genetic resources is crucial for the development of new red clover cultivars ensuring they better meet farmers’ changing needs and continue to benefit global pastoral farming industries.

Evaluating and efficiently characterizing large quantities of novel germplasm is hampered by cost and time requirements. One method is to develop core collections which involves selecting a representative subsample that encompasses the genetic diversity of the whole germplasm collection (Kouame and Quesenberry, 1993; Frankel, 1984). Approaches to developing core collections vary depending on the objectives and desired outcomes of individual programs, the tools available and the quality of passport data, such as country of origin, collection site abiotic and biotic features, and available phenotypic characteristics present in germplasm databases. Stratifying collections based solely on passport data to develop core collections is a cost-efficient method with much of the information readily available from germplasm databases (Brown, 1989). A simple tool for visualizing the amount of genetic diversity captured by this technique is to use a pedigree mapping tool. Pedigrees are useful for interpreting population structure and any potential genetic bottlenecks (Egan et al., 2019). However, this method is reliant on the quantity, quality and reliability of site information including history of introductions stored in germplasm databases and phenotypic data collected in the field. The continued advancement of molecular marker technologies such as genotyping-by-sequencing (GBS) have enabled researchers to characterize genetic diversity and population structure rapidly and cost-effectively within large collections of genetic material in forages (Nay et al., 2023; Faville et al., 2020). This facilitates screening of whole collections to develop a representative core collection but can still be costly when screening large collections.

A cost-effective germplasm stratification solution can be a combined approach where the collection is filtered using passport data to identify an initial eco-geographically diverse set of populations which can then be assessed for genetic diversity and population structure (Brown, 1989) derived from using methods such as pooled GBS (Faville et al., 2020). Essentially, this is GBS of a bulk of numerous individuals within a population and the resulting genetic data are derived from allele frequencies at each locus for the population as opposed to genotypes for individuals within the population (Blanco-Pastor et al., 2019). Bulk, or pooled, GBS is a cost-effective efficient way to access and harness genetic data especially for outbreeding, high genetic diversity crops such as red clover (Blanco-Pastor et al., 2019; Byrne et al., 2013; Faville et al., 2020). Previous studies have shown a pooled GBS technique can be effective in assessing genetic diversity amongst red clover populations (Zanotto et al., 2021; Frey et al., 2022; Nay et al., 2023 and Zanotto et al., 2023).

Assessing and utilizing red clover germplasm collections to bring new allele combinations into breeding programs has been applied rarely in red clover improvement. Previously, researchers have focused on evaluating genetic diversity among red clover germplasm populations predominantly from Northern Europe/Nordic backgrounds rather than in the warm temperate conditions where much red clover is grown (Zanotto et al., 2021, 2023; Osterman et al., 2021). The emphasis was on evaluating a selection of ecotypes and cultivars for improved yield, freezing tolerance, winter hardiness and survival (Zanotto et al., 2021, 2023; Osterman et al., 2021) or observing genetic diversity for key plant structural traits and persistence (Jones et al., 2020). More recently, Nay et al., 2023 explored genetic and phenotypic diversity of traits including dry matter yield and survival, amongst a more diverse selection of Southern and Central European red clover ecotypes. These studies did not include material from North Africa nor the southern Caucasus region including Turkey, Georgia, and Azerbaijan. Populations from these regions may contain important alleles for biotic adaption to heat and drought stresses which will likely be valuable for mitigating climate change effects, particularly in the temperate environments where much agronomic production occurs.

The relationships between environmental factors and the genetic variation present amongst populations, known as landscape genomics, is crucial for understanding the adaptative phenotypic characteristics present (Capblancq and Forester, 2021). By introducing germplasm populations into a new environment, such as common garden experiments, populations with higher local adaptation can be identified and compared to existing locally adapted populations (Capblancq et al., 2023). There is, however, a lack of studies that explore landscape genomics in red clover populations. The diverse geographical origins of the populations in this current study combined with genotype data will facilitate a better understanding of the relationship between environmental factors and plant adaptation, while also enabling the development of predictive models for plant performance and persistence within new environments (Capblancq and Forester, 2021). This provides another tool when selecting populations to form core collections or adding to existing breeding programs. Our aim was to identify and assess red clover material particularly from low rainfall areas that would add value to breeding programs by harnessing previously untapped genetic diversity from genebanks based on the following objectives which were to: (i) assess diverse red clover germplasm selected by eco-geographic passport data for insight into genetic diversity and population structure using pooled GBS, including alignment with pedigree data; (ii) characterize the populations in a grazed multi-site, multi-year field trial to determine quantitative genetic attributes of key agronomic traits for incorporation into breeding programs; and (iii) apply landscape genomics analyses to identify markers and genomic regions that may be implicated with field performance and adaptive traits.

## 2 Materials and methods

### 2.1 Plant material, trial design and trial management

Out of the 5223 red clover populations available in the Margot Forde Genebank at AgResearch Grasslands, Palmerston North, New Zealand, only 45% of populations had country of origin and collection site information available (Egan et al., 2019). Ecogeographical factors including both place of origin with latitude-longitude coordinates and collection site information along with seed availability were the main selection criteria for our panel of 92 populations. Increased weighting was given to selecting populations collected from low rainfall environments. The final selection of ecotypes was from the following countries: Armenia (Arm; 11 ecotypes); Azerbaijan (Azb; 8); Bosnia & Herzegovina (Bos; 1); Croatia (Cro; 1); Czech Republic (Cze; 1); Georgia (Geo; 5); Greece (Gre; 8); Italy (Ita; 6); Morocco (Mor; 1); Portugal (Por; 8); Russia (Rus; 12); Spain (Spa; 8); Tajikistan (Taj; 10); Turkey (Tur; 11); and United Kingdom (Uni; 1) (**Supplementary Table 1**). This comprised 15 countries with diverse eco-geographic data that represents the diversity of drier environments amongst the 41 countries with red clover seed samples stored at the Margot Forde Genebank (Egan et al., 2019).

To assess the agronomic potential of these diverse populations in contrasting environments, a field trial based on a row-column experimental design with two replicates was established in Spring 2020 at two sites, Lincoln (AgResearch Lincoln research farm: 43° 38’ S, 172° 30’ E) and Palmerston North, New Zealand (AgResearch Aorangi research farm: 40° 19’ S, 175° 29’ E). The Lincoln site was situated on Wakanui silt loam soil (Brown et al., 2005), 14 m above sea level. The annual rainfall for years 1, 2, 3 and for the years combined was 556.8 mm, 739.4 mm, 392.4 mm and 1688.6 mm respectively. Mean maximum temperature for years 1, 2, 3 and average overall was 18°C, 17°C, 20°C and 18°C, respectively. Mean minimum temperature for years 1, 2, 3 and average overall was 8°C, 8°C, 10°C and 8°C. The Palmerston North site was situated on Kairanga silt loam soil (Cowie, 1978), 15 m above sea level. The annual rainfall for years 1, 2, 3 and years combined was 1057.6 mm, 1371.5 mm, 540.4 mm and 2969.5 mm, respectively. Mean maximum temperature for years 1, 2, 3 and overall was 18°C, 18°C, 20°C and 19°C, respectively. Mean minimum temperature for years 1, 2, 3 and overall was 9°C, 10°C, 12°C and 10°C. Both locations were sown with a diploid perennial ryegrass (*Lolium perenne* L.) cultivar ‘Ceres One50’ containing the *Epichloë festucae* fungal endophyte strain AR37 to mimic a mixed sward cattle/sheep grazed management.

A random sample of seed from each red clover population was scarified using sandpaper, then germinated to provide sixteen plants per plot for each replicate. These plants were transferred individually to root trainer pots (3 cm x 3 cm x 10 cm) filled with Dalton’s Premium Potting Mix (Daltons^®^, Matamata, New Zealand). The plants were maintained in glasshouse conditions for two months (max./min. temperatures: 24°C/12°C; photoperiod: 9 h light/15 h dark). They were then transferred outside onto a concrete pad in late winter for “hardening” over two months to increase post-transplanting survival. In spring, field trials were established, sixteen plants of each population were hand transplanted into each plot. Plots were 1 m × 1 m with inter-plot spacings of 1.5 m. Within each plot, plants were arranged in four rows of four plants starting at the top left corner with approximately 25 cm between plants. Both trials were established in September (spring) 2020 and continued until March (early autumn) 2023. Prior to establishment, trial sites were treated with the selective herbicide Kamba^®^500 (Nufarm) for the control of volunteer clovers. During the life of the trial any volunteer clovers were spot-sprayed using Kamba500 as required. Soil tests were completed annually, and 15-20 kg ha^−1^ nitrogen in the form of Urea (46% w/w N) was applied twice a year at the Palmerston North site and once a year at the Lincoln site to maintain ryegrass health. At both locations, standard farming practices were followed by rotationally grazing the trials when herbage mass was between 2,500 and 2,800 kg DM ha^−1^, as assessed by a rising plate meter. Animals were removed when pasture residuals were between 1,000 to 1,200 kg DM ha^−1^. Each grazing was for a period of 1 to 2 days, season dependent, to ensure rapid and uniform defoliation and minimize the effect of urine and fecal patches in the trials. Trials were rotationally grazed by sheep at Lincoln and cattle at Palmerston North. Both sites were run as dryland with only natural precipitation for the entirety of the trial.

### 2.2 Morphological and physiological traits

Eight morphological and physiological traits were assessed at least seasonally each year. At both Lincoln and Palmerston North sites three key agronomic morphological traits were measured: (i) A qualitative visual score was used to measure plant biomass based on a 1 (low) to 9 (high) scale, with 1-unit increments. Calibration biomass cuts using a 0.25 m^2^ quadrat cutting to 4 cm and interspecies dissections were completed for each of the growth notes, with an average R^2^ alignment with visual scores of 0.81 and 0.67 at Lincoln and Palmerston North, respectively. (ii) Growth habit was assessed using a qualitative visual 1 to 4 scale with 1 = erect habit, 2 = semi-erect habit, 3 = semi-prostrate, 4 = prostrate. (iii) Average leaf size per plot was measured using a qualitative visual 1 (low) to 5 (high) scale, with 1-unit increments. As Lincoln was used as a common garden experiment site five further traits were measured: (i) A qualitative visual score was used to measure plant plot density based on a 1 (low) to 9 (high) scale, with 1-unit increments. (ii) Average plot height (cm) was measured using a ruler at the highest three points of the plot; (iii) Plant survival was a visual count of survivors within each plot of 16 plants. (iv) The average lamina area of two randomly chosen young, fully unfolded leaves per plant was determined using the open-source image processing and analysis program *“ImageJ”* (Katabuchi, 2015). (v) Relative chlorophyll content was measured using a Konica Minolta SPAD-502Plus chlorophyll meter. An average reading was taken from three randomly selected young, fully unfolded leaves per plant (Ling et al., 2010). All traits were measured immediately prior to grazing.

### 2.3 DNA isolation and genotyping of bulked samples

For DNA extraction, a random sample of each of the accessions was germinated and grown to the four-leaf stage in glasshouse conditions as described above. Sample harvesting and DNA extraction for bulked samples of each population was performed as described previously for white clover (Faville et al., 2020). Briefly, a single trifoliate leaf was harvested from thirty individuals for each red clover population germinated as described above and combined into a single bulked sample. To ensure similar representation of each individual within a population, effort was made to maintain similarity of harvested leaf size for each individual in the bulk. The leaf material for each population was freeze-dried, ground to a fine powder and DNA extracted as described (Anderson et al., 2018). Genotyping-by-sequencing (GBS) libraries were developed in 96-plex using a protocol adapted from Griffiths et al. (2019) with modifications as described by Faville et al. (2020) including digestion with *Pst* I restriction enzyme and inclusion of four technical replicates for each population. Each library was single-end sequenced (150 bp reads) on two lanes of an Illumina HiSeq 2500 (Illumina, San Diego, CA, USA) at AgResearch Invermay, New Zealand. Single nucleotide polymorphisms (SNPs) were identified using a custom SNP identification pipeline. In short, reads were quality-checked, demultiplexed, and mapped to the ARS RC 1.1 (GCA_020283565.1) red clover reference genome from Bickhart et al. (2022) using *“Burrows Wheeler Aligner”* v0.7.17 (Li and Durbin, 2009). SNP variants were detected using the *“mpileup”* command from *“bcftools”* v1.13 (Li, 2011), filtering low mapping quality (MQ>20) and base quality (BQ>20) reads. *“Vcftools”* v0.1.16 (Danecek et al., 2011) then utilized to only include the SNPs with coverage across greater than 95% of samples, allele frequencies between 0.05 and 0.95 in at least 10 populations, and mean allele frequencies across all populations between 0.05 and 0.95. Allele frequencies were combined across the four technical replicates and were an estimate of the allele frequency at a SNP for the pooled sample. Allele frequencies were extracted and frequencies of one indicated homozygosity of the population for the reference allele and an allele frequency of zero indicated homozygosity of the alternative allele. The alternative allele frequency (AAF) was calculated by using the following equation:


AAF=Alt/(Alt+Ref)

Where: Alt is the alternative allele count and Ref is the reference allele count (Li, 2011). For analysis, missing data of the filtered SNPs with greater than 95% coverage was replaced by mean allele frequency across populations at the given SNP position. SNP filtering and missing data replacement was completed using a custom R script which is available on GitHub.

### 2.4 Population structure and genomic relationship matrix

Modified Rogers Distance was used to calculate genetic distance between different genotypes using the alternative allele frequency data of the 92 populations. An initial optimal number of principal components to apply to the discriminant analysis of principal components (DAPC) analysis was determined by generating an A-score (Jombart et al., 2018). The optimal number of PCs was incorporated into the K-means clustering algorithm which was run iteratively from 1 to 85 genetic clusters (K). The optimal cluster number corresponded with the lowest Bayesian information criterion value. DAPC (Jombart et al., 2010) was performed using *“adgenet”* v2. 1 (Jombart, 2008) to assess population structure and individuals were then assigned to genetic clusters. Analysis of molecular variance between the identified genetic clusters, countries of origin and amongst the 92 populations and fixation index (F_ST_) values were calculated using the *“poppr”* and *“hierfstat”* R packages respectively (Kamvar et al., 2014; De Meeûs and Goudet, 2007). To interpret F_ST_ values we used Wright (1978) suggestion for biallelic markers with little genetic differentiation (0-0.05), moderate genetic differentiation (0.05-0.15), and great genetic differentiation (0.15-0.25) (Cheng et al., 2020). Expected heterozygosity (*H*_E_) or gene diversity of the clusters were also calculated, and *H*_E_ values computed using the *“dplyr”* R package (Wickham et al., 2023). Geneflow was calculated using the following equation:


$$Nm=\frac{1-F_{ST}}{4*F_{ST}}$$

Where: Nm is gene flow and F_ST_ is the fixation index value. To assess gene flow levels, we used Wright’s (1978) classification of three grades: low gene flow (0.0 to <0.25), medium gene flow (0.25 to 0.99) and high geneflow (>1.0) (Cheng et al., 2020). As the pooled samples contained multiple genomes, alleles were combined by multiplying alternative allele frequencies by two to fit a “0/0, 0/1, 1/1” structure then recoded to a -1 to 1 scale to generate a genomic-based relationship matrix. The genomic based relationship matrix was generated based on the method proposed by VanRaden (2008) using the *A.mat* function in the R package *“rrBLUP”* (Endelman, 2011). A dendrogram was generated using the *“Ape”* R package (Paradis et al., 2004).

### 2.5 Pedigree information and relationship matrix

Pedigree information for 77 of the 92 populations was extracted from a database previously established by Egan, et al. (2019), where they constructed a pedigree map of the entire red clover collection of the Margot Forde Genebank. A pedigree-based relationship matrix (A matrix) was generated and displayed as a heatmap using the *“ASRgenomics”* tool and *“AsReml-R”* R package (Butler et al., 2017). The R package *“pedigree”* was used to calculate kinship and inbreeding as shown in Egan et al. (2019).

### 2.6 Variance components and heritability

As the 92 populations were not part of a structured population, the genomic relationship matrix information was used to enable estimation of quantitative parameters including narrow-sense heritability. A linear mixed model was fitted for the eight morphological and physiological traits using *“AsReml-R”* v4.2 (Butler, et al., 2017). The following linear mixed model was fitted as a full model and altered depending on the analysis needed using *“AsReml-R”*:


$$Y_{inojklm}=\;\mu +p_{i}+y_{o}+e_{n}+{\left(pe\right)}_{in}+s_{j}+{\left(ps\right)}_{ij}+{\left(py\right)}_{io}+{\left(sy\right)}_{jo}+{\left(pey\right)}_{ino}+{\left(psy\right)}_{ioj}+b_{nojk}+r_{nojkl}+c_{nojkm}+e_{inojklm}$$

Where: *Y_inojklm_* is the value of a trait measured from population *i*, at the *n*th location of the *o*th year in the *j*th season, within the *k*th replicate, in *l*th row, and *m*th column. *i* = 1,…, *n_f_*; *n* = 1,…, *n_e_*; *o* = 1,…, *n_y_*, *j* = 1,…, *n_s_*; *k* = 1,…, *n_b_*; *l* = 1,…, *n*_r_; *m* = 1,…, *n_c_*; where, *p*, *y*, *e*, *s*, *b*, *r*, and *c* are populations, years, locations, seasons, replicates, rows, and columns, respectively. *µ* is the overall mean; *p*_i_ is the random effect of population *i* and distributed as *N*(0, *G*σ^2^ *^g^*); where *G* is the genomic relationship matrix, *y_o_* is the fixed effect of year *o*; *e_n_* is the fixed effect of location *n*; (*pe*)*_in_* is the random effect of the interaction between population *i* and location *n*, *N*(0, *I*σ^2^_ge_); where *I* is the identity matrix, *s_j_* is the fixed effect of season *j*, *N*(0, *I*σ^2s^); (*ps*)_ij_ is the random effect of the interaction between population *i* and season *j*, *N*(0, *I*σ^2gs^); (*py*)*_io_* is the random effect of the interaction between population *i* and year *o*, *N*(0, *I*σ^2io^); (*sy*)*_jo_* is the random effect of the interaction between season *j* in year *o*, *N*(0, *I*σ^2^ *^sy^*); (*pey*)*_ino_* is the random effect of the interaction between population *i* location *n* and year *o*, *N*(0, *I*σ^2^ *^gey^*); (*psy*)*_ioj_* is the random effect of the interaction between population *i* season *j* and year *o*, *N*(0, *I*σ^2^ *^ioj^*); *b_nojk_* is the random effect of replicate *k* within season *j* within year *o* in location *n*, *N*(0, *I*σ^2^ *^b^*); *r_nojkl_* is the random effect of row *l* within replicate *k* in season *j* within year *o* in location *n*, *N*(0, *I*σ^2r^); *c_nojkm_* is the random effect of column *m* in replicate *k* within season *j* within year *o* in location *n*, *N*(0, *I*σ^2c^); and *e_inojklm_* is the residual effect of population *i* in location *n* within year *o* in season *j* of replicate *k* in row *l* and column *m* of replicate *k* in season *j* within year *o* in location *n*. This produced residual maximum likelihood estimates of the variance components for each trait and genomic best linear unbiased prediction values for each population.

The variance components estimated on the linear mixed model analysis were used to calculate a narrow sense heritability or genomic heritability based on G-matrix for each trait using different adaptations of the following equation:


$$h_{n}^{2}=\;\frac{\sigma_{p}^{2}}{\left(\sigma_{p}^{2})+(\sigma_{pb}^{2})/n_{r}+(\sigma_{ps}^{2})/n_{s}+(\sigma_{py}^{2})/n_{y}+(\sigma_{pl}^{2})/n_{l}+(\sigma_{e}^{2})/(2*4*3*2\right)}$$

Where: *h^2n^* is narrow sense heritability. In the estimation of narrow sense heritability; σ^2p^ was the estimated additive genetic variation amongst the 92 populations; σ^2pb^ is the variance associated with the population-by-replicate interaction; n_r_ was the number of replicates; σ^2ps^ is the variance associated with population-by-season interaction; n_s_ was the number of seasons; σ^2py^ is the variance associated with population-by-year; n_y_ was the number of years; σ^2pl^ is the variance associated with population-by-location interaction; n_l_ was the number of years and σ^2e^ is the variance of residuals (Arojju et al., 2020). From here on, heritability will be referred to as narrow sense heritability.

### 2.7 Landscape genomic analysis

Redundancy analysis was performed using the *“Vegan”* package in RStudio, with morphological and physiological traits as the response variable and bioclimatic variables the explanatory variable (Capblancq and Forester, 2021; Oksanen et al., 2013). Based on collection site coordinates obtained from passport information of the 92 populations retrieved from the Margot Forde Genebank database, the analysis used 19 bioclimatic variables (Bio1 to Bio19) sourced from the ‘WorldClim’ database (*worldclim.org*) (**Supplementary Table 1**). Bioclimatic variables were extracted at 2.5 arcminutes (~4.5 km at the equator) spatial resolution (Hijamans et al., 2005). A superior model was fitted after multiple models were run, containing variables that explained the most variation present and while also reducing collinearity through removing highly correlated (*r* ≥ 0.8) variables based on the Pearson correlation coefficient. Biplots were generated to show associations between the environmental explanatory factors, cluster groupings, explanatory (plant performance) and trait response by using the *“ggplot2”* R-package and the *“Data integration app”* (Wickham, 2011; Luo, 2022).

Candidate SNPs, SNPs that showed significant associations with environmental variables, involved in local adaptation, were identified through redundancy analysis using the *“Vegan”* package in RStudio (Oksanen et al., 2013; Capblancq and Forester, 2021). Genetic information from the filtered SNPs of the 92 populations served as the response variables, while bioclimatic variables were the explanatory variables. Candidate SNPs were identified has having a threshold of 3 standard deviations from the mean SNP loading, a false discovery rate correction applied to adjust for multiple comparisons and to avoid false discoveries (Benjamini and Hochberg, 1995). For each candidate SNP the bioclimatic variables most strongly correlated with it was identified along with *p*-values for associations. Using the ARS RC 1.1 (GCA_020283565.1) red clover reference genome (Bickhart et al., 2022), the closest gene associated with each SNP was extracted using a custom R script. Gene descriptions including type and role and the location of the SNP in reference to the allele were also collated.

Gene Ontology (GO) terms for the associated genes for the candidate SNPs involved in local adaptation were annotated using the package *“GOMAP‐Singularity”* v1.3.4 (Wimalanathan and Lawrence-Dill, 2021) with the default parameters. The GO term information was then sourced from the NCBI database. GO term enrichment was performed using the *“topGO R‐package”* v2.54.0 (Alexa, 2023) for the molecular function process ontology with the ‘weight01’ algorithm and Fisher’s exact test. Enriched GOterms were filtered based on a p‐value threshold of < 0.05. The GO enrichments were visualized as treemaps generated using *“rrvgo”* v1.14.1 (Sayols, 2023) with the *Trifolium pratense* annotation database (Sayers et al., 2022). The scores were set as the p‐values resulting from the topGO enrichment analysis. A chi-square test was performed to assess independence between the SNP frequency and cluster membership, and a false discovery rate correction was applied to adjust for multiple comparisons. All statistical analysis were performed in R v4.4.1 (R Core Team, 2021), implemented in Rstudio (RStudio Team, 2020).

## 3 Results

### 3.1 Genetic diversity of global red clover populations showed geographic clustering

**Figure 1:**
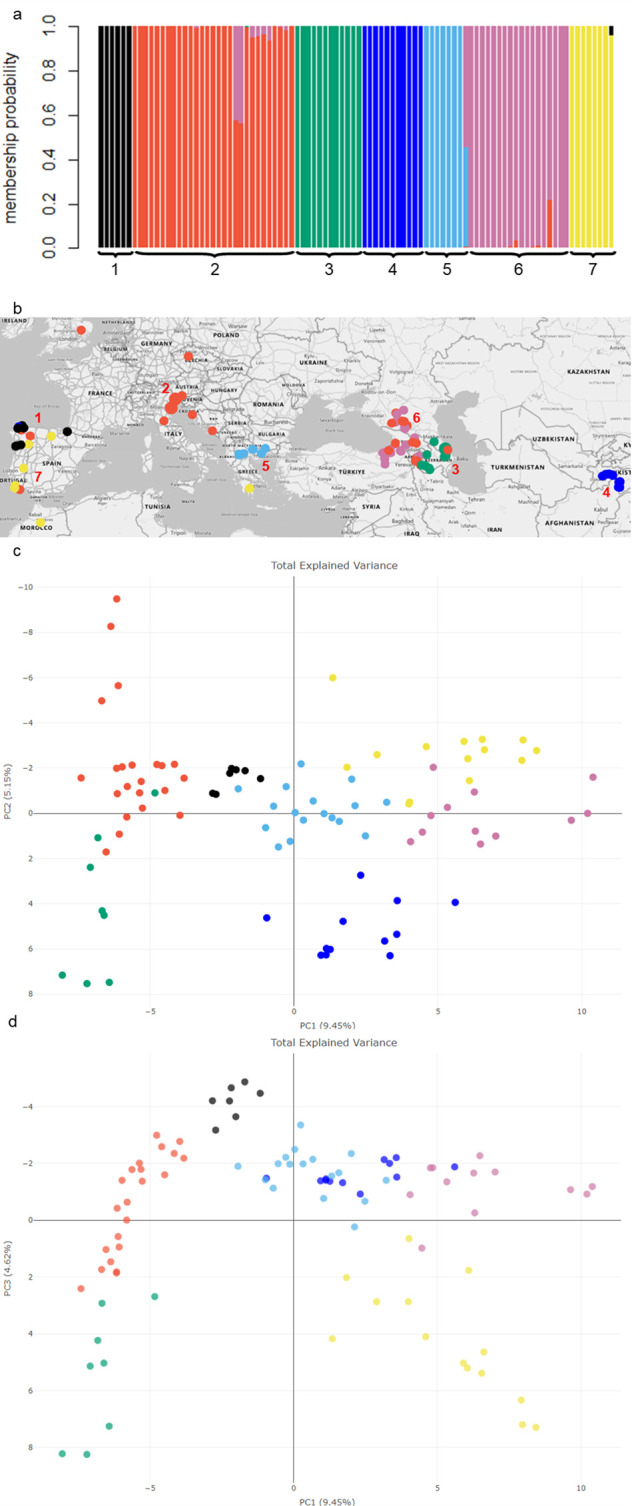
Population structure of 92 red clover populations and their alignment with geographic origins **(a)** Membership probability based on an optimum of seven clusters using DAPC analysis **(b)** The geographic location of each germplasm population along with color coordinated cluster groupings. The map was generated using Microsoft Excel (Microsoft Corporation, 2024), with geospatial data sourced from Bing Maps. **(c)** Principal component analysis plot based on Modified Rogers Distance among the 92 red clover populations showing components PC1 and PC2 and **(d)** components PC1 and PC3. Populations have been assigned to the seven genetic clusters indicated by color.

GBS of pooled samples for each of the 92 red clover populations identified 12,168 SNPs which reduced to 4,509 SNPs after filtering and were then used to construct a genomic relationship matrix for the 92 populations. The A-score optimization determined that seven principal components were ideal (**Supplementary Figure S1**) for the DAPC analysis which indicated two to eight clusters as potential outcomes based on Bayesian Information Criteria values (**Supplementary Figures S2**, **S3**). Assessing the different grouping options with the geographic information identified seven clusters as an optimal interpretation of the data, as these clusters predominantly aligned with their geographical sources (**Figure 1**; **Supplementary Table S2**). Cluster membership probability (**Figure 1a**) showed little admixture among the populations, although there was some evidence of mutual geneflow where clusters overlapped geographically such as Clusters 2 and 6 in the western Caucasus, and Clusters 1 and 7 in the northern Iberian Peninsula (**Figures 1a, b**). Geographic placement of the groupings showed Clusters 4 and 5 were the most localized and contained populations restricted either to Tajikistan (with one exception in northern Spain), or northern Greece, respectively (**Figure 1b**). The Caucasus region had two distinct groups, Cluster 3 in the east (Armenia, Azerbaijan, and Caucasian Russia), and Cluster 6 in the west (Armenia, Georgia, Caucasian Russia, and Eastern Turkey), as well as an overlap with the cosmopolitan Cluster 2 which encompassed the Caucasus, Central Europe to the United Kingdom. Cluster 1 was restricted to northern Spain/Portugal and co-located with some members of Clusters 2, 4 and particularly 7 which contained populations from northern Spain through Portugal, North Africa (Morocco) and southern Greece (**Figure 1b**).

The principal components PC1, PC2 and PC3 together explained 19.22% of the genetic variation (**Figure 1c**). Separation of clusters along PC1 (9.45%) aligned with longitude as the samples following a transect from Central Asia (Tajikistan) to Western Europe, whereas PC2 (5.15%) appeared to be driven by variation in the Iberian Peninsula (Clusters 1 and 7 in particular) and may represent differences in latitude (**Figure 1c**). PC3 (4.62%) separated clusters in the Iberian Peninsula further from the rest, similarly with the Greek cluster and Tajikistan populations (**Figure 1d**).

### 3.2 Genetic diversity found to be greatest amongst populations with high gene flow

Analysis of molecular variance was used to characterise the total genetic variation present between clusters and among populations (**Table 1**). Of the three levels of variation assessed, variation amongst the seven clusters and country of origin accounted for 14.6% and 6.4% of the total variance, respectively. The greatest source of variance (79.1%) was detected among populations. Pairwise F_ST_ values (**Table 2**) showed moderate differentiation between most of the clusters with the highest value (0.098) being between Clusters 1 (Northern Spain/Portugal) and 4 (predominantly Tajikistan) which are geographically the most distant. A single population belonging to Cluster 4 that was located in Northern Spain rather than Tajikistan is a potential artefact due to mislabeling in either the germplasm center or in the laboratory workflow. Of the seven clusters, four had pairwise F_ST_ values between 0.037 and 0.044, indicating little genetic differentiation. These were either geographically adjacent clusters, such as Clusters 3 and 6 (both Caucasus) and Clusters 4 and 6 (Tajikistan and Caucasus) or overlapped with the region as exemplified by the pairwise F_ST_ of Clusters 3 and 6 with the cosmopolitan Cluster 2 (**Table 2**; **Figure 1c**). These relationships were reflected in the geneflow estimates which ranged from 2.3 to 6.5 and indicated high geneflow (≥ 1.0) among the clusters, particularly with increasing geographic proximity (**Table 2**).

**Table 1:** Results of an analysis of molecular variance between the seven red clover clusters, countries of origin and amongst populations.

Source of variation	Degrees of freedom	Sum of Squares	Mean Square	Variance explained (%)	F-value	P-value
Clusters	6	26430	4405	14.6	3.12	0.001
Countries of Origin	21	29631	1411	6.4	0.08	0.001
Populations	64	75255	1176	79.1		0.001
Total	91	131316	1443	100		

**Table 2:** Results of pairwise F_ST_ calculations (lower triangle) and Geneflow (upper triangle) among the seven red clover clusters.

	Cluster1	Cluster2	Cluster3	Cluster4	Cluster5	Cluster6	Cluster7
**Cluster1**	~	3.85	2.35	2.30	2.53	2.66	3.18
**Cluster2**	0.061	~	5.46	4.21	3.85	6.51	3.85
**Cluster3**	0.096	0.044	~	3.43	2.80	5.70	2.84
**Cluster4**	0.098	0.056	0.068	~	2.53	5.70	2.38
**Cluster5**	0.090	0.061	0.082	0.090	~	3.43	2.96
**Cluster6**	0.086	0.037	0.042	0.042	0.068	~	2.84
**Cluster7**	0.073	0.061	0.081	0.095	0.078	0.081	~

**Figure 2:**
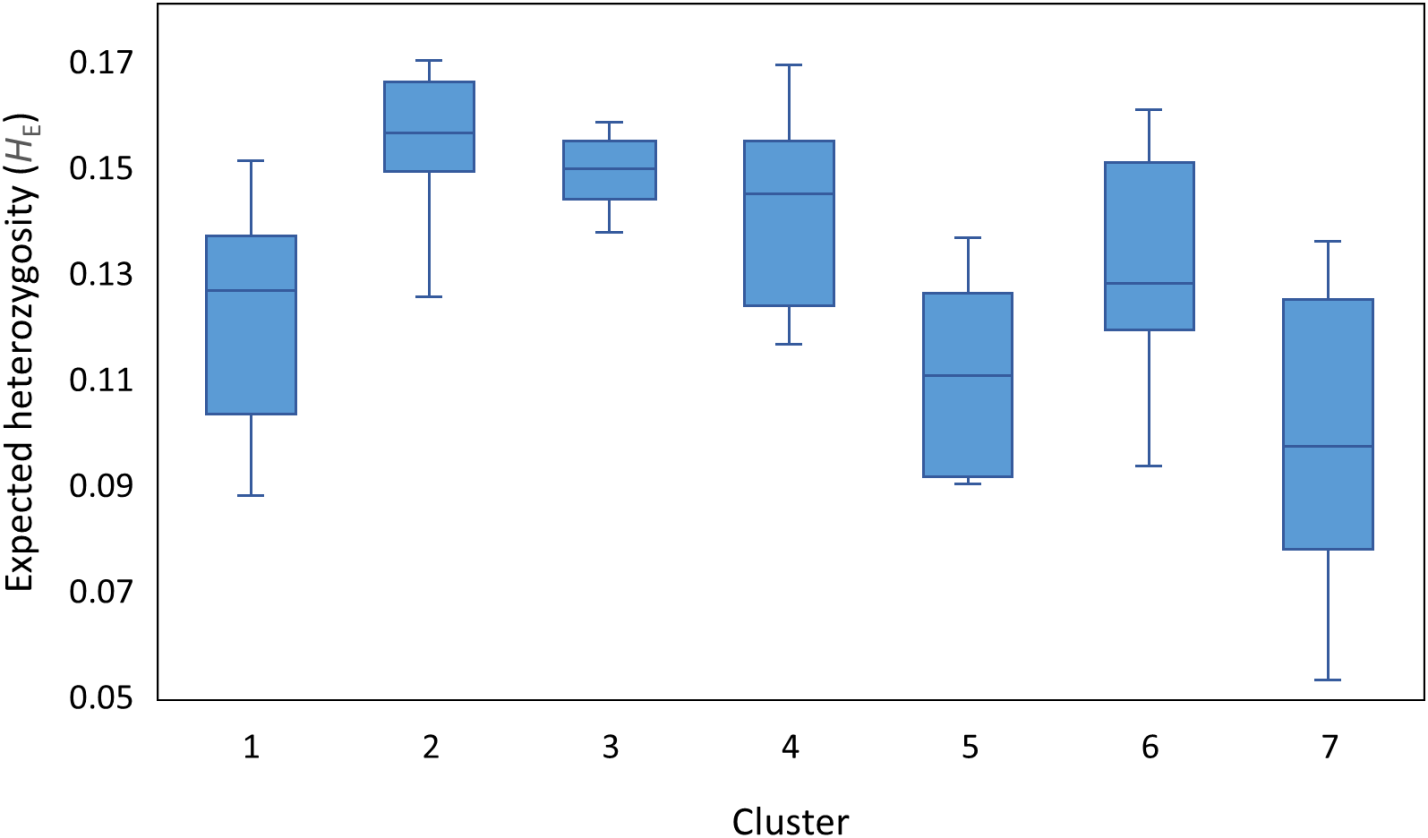
Expected heterozygosity (*H*_E_) to assess genetic diversity within the seven red clover clusters identified by DAPC genetic diversity analysis.

Expected heterozygosity (*H*_E_) was calculated to assess genetic variation within each of these clusters (**Figure 2**). The mean *H*_E_ ranged from 0.10 to 0.16 with the Iberian Peninsula/North African/Southern Greece-derived Cluster 7 having the lowest mean *H*_E_ but also exhibited the greatest variation in *H*_E_. By contrast, the cosmopolitan Cluster 2 and the Eastern Caucasus-based Cluster 3 exhibited the greatest *H*_E_ values as well as the least variability among their constituent populations (**Figure 2**). Clusters 1, 4, 5 and 6 had similar amounts of variation amongst populations.

### 3.3 Genotype data provided more insight than pedigree information

**Figure 3:**
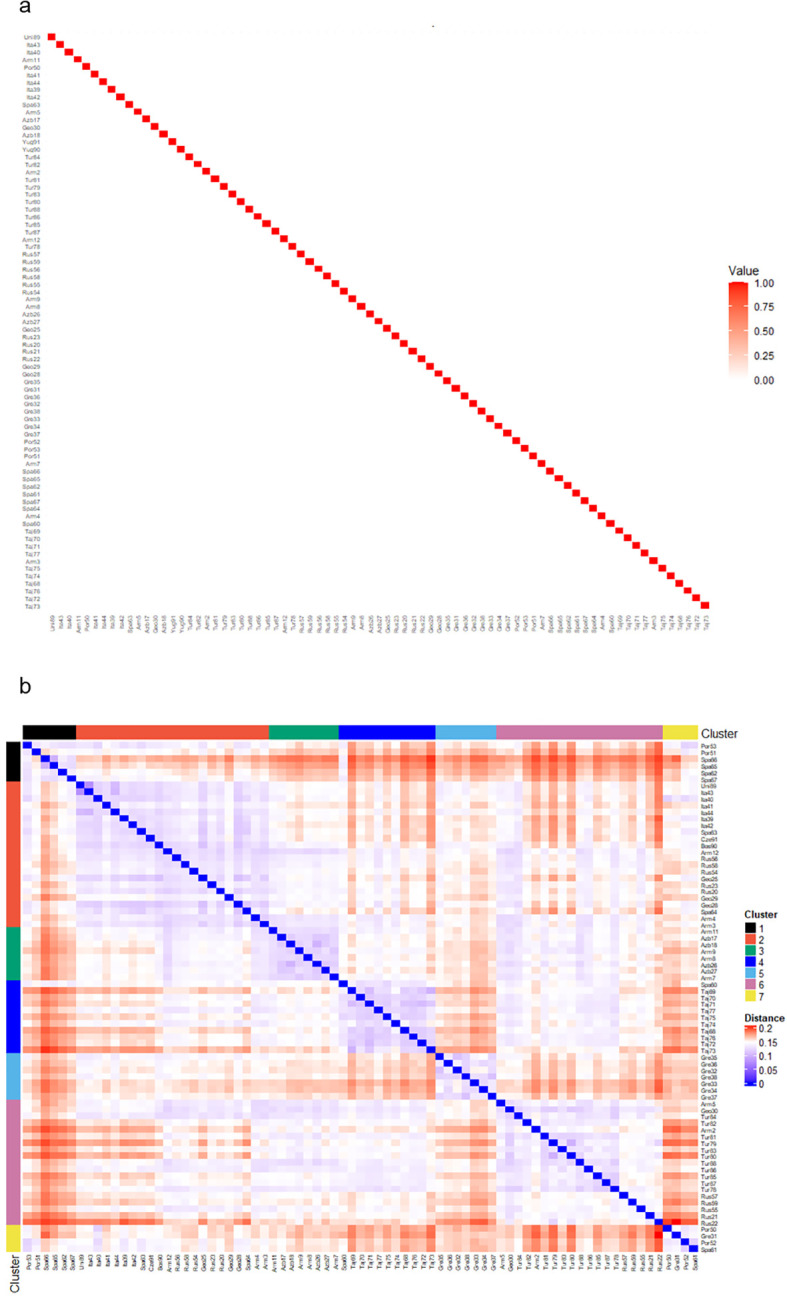
Comparison of interpopulation relationships based on either pedigree or genotype information. **(a)** A heatmap of the pedigree relationship matrix (A-matrix) of the 77 populations. As most accessions are founders with no pedigree details, no A-matrix was produced, hence only accession relationships with themselves shown. **(b)** Heatmap of the genetic relationship matrix for the 77 populations based on the Modified Roger’s distances. Accessions are ordered by groups based on discriminant analysis of principal components (DAPC) analysis cluster analysis.

Comparisons between genetic and pedigree matrices for the 77 populations with available pedigree information are shown in **Figure 3**. As most of these populations were classified as founders, we were unable to estimate inter-population relationships based on pedigree data, hence a pedigree relationship matrix (A-matrix) could not be calculated (**Figure 3a**). This reflected that the populations had no prior breeding history or generational structure, but did, however, suggest a lack of inbreeding within or relatedness among populations. By contrast, the GBS-derived genotype data identified clear structures of relationships and groupings between the 77 populations as indicated through color differentiation and clades on the modified Roger’s distance heatmap that were undetectable using the pedigree data (**Figure 3b**).

### 3.4 Significant variance components and narrow-sense heritability identified

When assessing the 92 populations in the field, population by locations interactions were highly significant (*p ≤* 0.001) for biomass, growth habit and leaf size across years and seasons (**Tables 3**, **4**). Across both sites and at the Lincoln site there were varying significant (*p* < 0.01) population by season interactions (σ^2P×S^) and population by year interactions (σ^2PxY^) for biomass, growth habit, leaf size, and additionally at the Lincoln site for plot height, chlorophyll content and plot density (**Tables 5**, **6**). At the Palmerston North site, only year 1 biomass had a highly significant (*p ≤* 0.001) population by season interaction and only spring and summer biomass had highly significant (*p ≤* 0.001) population by year interactions. As the population significant variance was greater than the population by location variance this indicated that the performance of the populations was relatively consistent between sites.

**Table 3:** Estimated population (σ^2<sub>P</sub>^), population-by-season (σ^2<sub>P×S</sub>^), population-by-location (σ^2<sub>P×E</sub>^), population-by-year (σ^2<sub>P×Y</sub>^), residual error (σ^2<sub>Er</sub>^); variance components, their associated interactions, and standard errors (± SE); and population mean narrow-sense heritability (*h^2^*) for biomass, growth habit and leaf size for 92 red clover germplasm populations across two locations, Lincoln and Palmerston North, and per year and across 3 years. Significance levels of interaction (*P < 0.05; **P < 0.01; *** P < 0.001).

Source of variation	Biomass			Growth habit			Leaf size		
	Lincoln	Palmerston North	Combined	Lincoln	Palmerston North	Combined	Lincoln	Palmerston North	Combined
Across years									
σ^2P^	1.12 ± 0.21	1.16 ± 0.26	0.86 ± 0.20	0.20 ± 0.04	0.18 ± 0.05	0.16 ± 0.03	0.40 ± 0.07	0.37 ± 0.07	0.38 ± 0.07
σ^2P×S^	0.24 ± 0.06 ***	0.18 ± 0.07 ns	0.10 ± 0.03 ***	0.02 ± 0.01 ***	0.00 ns	0.02 ± 0.01 ***	0.06 ± 0.01 ***	0.00 ns	0.03 ± 0.01 ***
σ^2Er^	1.8 ± 0.1	2.57 ± 0.11	2.20 ± 0.06	0.29 ± 0.01	0.53 ± 0.05	0.36 ± 0.02	0.42 ± 0.02	0.32 ± 0.03	0.42 ± 0.02
σ^2P×E^	~	~	0.26 ± 0.06 ***	~	~	0.03 ± 0.01 ***	~	~	0.02 ± 0.01 ***
σ^2P×Y^	0.19 ± 0.05 ***	0.44 ± 0.11 ns	0.32 ± 0.06 ***	0.02 ± 0.01 ***	0.00 ns	0.00 ns	0.03 ± 0.01 ***	0.00 ns	0.04 ± 0.01 ***
*h^2^*	0.81	0.74	0.7	0.87	0.68	0.76	0.84	0.77	0.85
Year 1									
σ^2P^	1.31 ± 0.27	1.52 ± 0.35	1.44 ± 0.27	0.23 ± 0.04	0.09 ± 0.06	0.13 ± 0.04	0.44 ± 0.08	0.40 ± 0.09	0.45 ± 0.08
σ^2P×S^	0.58 ± 0.14 ***	0.67 ± 0.19 ***	0.04 ± 0.07 ***	0.00 ns	~	~	0.02 ± 0.03 ***	~	0.00 ns
σ^2Er^	1.70 ± 0.13	1.94 ± 0.17	2.09 ± 0.11	0.25 ± 0.02	0.45 ± 0.06	0.35 ± 0.03	0.41 ± 0.03	0.29 ± 0.04	0.42 ± 0.03
σ^2P×E^	~	~	0.15 ± 0.08 ***	~	~	0.00 ns	~	~	0.00 ns
*h^2^*	0.76	0.72	0.82	0.84	0.26	0.55	0.84	0.68	0.86
Year 2									
σ^2P^	1.68 ± 0.32	1.77 ± 0.33	1.08 ± 0.25	0.17 ± 0.04	0.38 ± 0.11	0.15 ± 0.04	0.37 ± 0.07	0.41 ± 0.10	0.36 ± 0.06
σ^2P×S^	0.00 ns	0.00 ns	0.02 ± 0.06 ***	0.00 ns	~	0.04 ± 0.03 ns	0.00 ns	~	0.00 ns
σ^2Er^	2.06 ± 0.14	2.69 ± 0.14	2.42 ± 0.11	0.28 ± 0.02	0.47 ± 0.07	0.38 ± 0.03	0.39 ± 0.03	0.34 ± 0.05	0.38 ± 0.02
σ^2P×E^	~	~	0.58 ± 0.13 ***	~	~	0.01 ± 0.03 ns	~	~	0.00 ns
*h^2^*	0.8	0.79	0.66	0.73	0.61	0.64	0.81	0.71	0.88
Year 3*									
σ^2P^	1.01 ± 0.24	1.59 ± 0.89	1.10 ± 0.28	0.28 ± 0.06	~	~	0.55 ± 0.12	~	~
σ^2P×S^	0.54 ± 0.13 ***	0.00 ns	0.35 ± 0.12 ***	0.06 ± 0.03 ***	~	~	0.16 ± 0.05 ***	~	~
σ^2Er^	1.05 ± 0.08	3.21 ± 0.69	1.38 ± 0.10	0.33 ± 0.03	~	~	0.46 ± 0.04	~	~
σ^2P×E^	~	~	0.25 ± 0.15 **	~	~	~	~	~	~
*h^2^*	0.63	0.44	0.63	0.75	~	~	0.72	~	~

Significance levels of interaction (*P< 0.05; **P< 0.01; *** P< 0.001).

**Table 4:** Estimated population (σ^2<sub>P</sub>^), population-by-location (σ^2<sub>PxE</sub>^), population-by-year (σ^2<sub>PxY</sub>^), residual error (σ^2<sub>Er</sub>^); variance components, their associated interactions, and standard errors (± SE); and population mean narrow-sense heritability (*h^2^*) for biomass, growth habit and leaf size for 92 red clover germplasm populations across two locations, Lincoln, and Palmerston North, and over 3 seasons.

Source of variation	Biomass			Growth habit			Leaf size		
	Lincoln	Palmerston North	Combined	Lincoln	Palmerston North	Combined	Lincoln	Palmerston North	Combined
Spring									
σ^2P^	1.27 ± 0.25	1.12 ± 0.35	1.01 ± 0.23	0.23 ± 0.05	0.38 ± 0.11	0.16 ± 0.04	0.51 ± 0.09	0.40 ± 0.10	0.42 ± 0.08
σ^2Er^	1.31 ± 0.09	2.69 ± 0.3	1.92 ± 0.09	0.28 ± 0.02	0.47 ± 0.07	0.33 ± 0.02	0.37 ± 0.02	0.29 ± 0.04	0.51 ± 0.03
σ^2P×Y^	0.43 ± 0.11 ***	0.34 ± 0.28 ***	0.31 ± 0.08 ***	0.03 ± 0.02 ns	0.00 ns	0.04 ± 0.02 *	0.06 ± 0.02 ***	0.00 ns	0.01 ± 0.03 ***
σ^2P×E^	~	~	0.34 ± 0.10 ***	~	~	0.04 ± 0.02 **	~	~	0.00 ns
*h^2^*	0.73	0.55	0.66	0.79	0.61	0.62	0.82	0.68	0.88
Autumn									
σ^2P^	1.05 ± 0.23	1.79 ± 0.36	1.14 ± 0.24	0.17 ± 0.04	~	~	0.46 ± 0.08	0.41 ± 0.10	0.42 ± 0.08
σ^2Er^	2.10 ± 0.18	2.88 ± 0.18	2.58 ± 0.13	0.31 ± 0.03	~	~	0.56 ± 0.04	0.34 ± 0.05	0.36 ± 0.02
σ^2P×Y^	0.22 ± 0.16 ***	0.00 ns	0.11 ± 0.08 ***	0.02 ± 0.02 ***	~	~	0.00 ns	0.00 ns	0.07 ± 0.02 ***
σ^2P×E^	~	~	0.18 ± 0.09 ***	~	~	~	~	~	0.05 ± 0.03 ***
*h^2^*	0.67	0.75	0.72	0.68	~	~	0.81	0.71	0.8
Summer									
σ^2P^	1.68 ± 0.34	1.20 ± 0.34	0.87 ± 0.26	0.29 ± 0.06	0.09 ± 0.06	0.24 ± 0.05	0.38 ± 0.07	~	~
σ^2Er^	1.65 ± 0.17	2.28 ± 0.17	1.90 ± 0.13	0.25 ± 0.02	0.45 ± 0.06	0.38 ± 0.03	0.28 ± 0.02	~	~
σ^2P×Y^	0.01 ± 0.15 ***	0.59 ± 0.21 ***	0.27 ± 0.11 ***	0.00 ns	0.00 ns	0.00 ns	0.00 ns	~	~
σ^2P×E^	~	~	0.58 ± 0.15 ***	~	~	0.00 ns	~	~	~
*h^2^*	0.8	0.58	0.57	0.82	0.26	0.71	0.84	~	~

Significance levels of interaction (*P< 0.05; **P< 0.01; *** P< 0.001).

**Table 5:** Estimated population (σ^2<sub>P</sub>^), population-by-season (σ^2<sub>P×S</sub>^), population-by-year (σ^2<sub>P×Y</sub>^), residual error (σ^2<sub>Er</sub>^); variance components, their associated interactions, and standard errors (± SE); and population mean narrow-sense heritability (*h^2^*) for plot height, plot density, chlorophyll content, lamina area and survival for 92 red clover germplasm populations at Lincoln per year and across 3 years.

Source of variation	Plot height	Plot Density	Chlorophyll content	Lamina area	Survival
Across years					
σ^2P^	8.83 ± 1.87	1.14 ± 0.21	3.67 ± 0.78	3.22 ± 0.73	4.61 ± 0.90
σ^2P×S^	6.56 ± 0.97 ns	0.03 ± 0.04 ***	0.01 ± 0.32 ***	0.74 ± 0.38 ns	0.00 ns
σ^2Er^	13.98 ± 0.63	1.80 ± 0.07	12.87 ± 0.63	3.24 ± 0.34	6.54 ± 0.38
σ^2P×Y^	0.11 ± 0.32 ***	0.26 ± 0.06 ***	0.18 ± 0.35 ***	~	0.65 ± 0.34 ***
*h^2^*	0.63	0.84	0.7	0.72	0.73
Year 1					
σ^2P^	11.10 ± 2.43	1.09 ± 0.23	4.50 ± 1.09	6.57 ± 1.47	0.13 ± 0.24
σ^2P×S^	6.12 ± 1.39 ***	0.37 ± 0.13 ***	0.00 ns	0.00 ns	0.00 ns
σ^2Er^	12.80 ± 1.11	1.75 ± 0.13	12.13 ± 0.81	4.27 ± 0.62	2.55 ± 0.33
*h^2^*	0.69	0.71	0.57	0.75	0.09
Year 2					
σ^2P^	8.97 ± 2.16	1.68 ± 0.32	3.34 ± 0.90	1.96 ± 0.48	6.98 ± 1.44
σ^2P×S^	6.38 ± 1.60 ***	0.00 ns	0.00 ns	0.00 ns	0.00 ns
σ^2Er^	15.45 ± 1.36	1.95 ± 0.13	11.61 ± 0.78	1.92 ± 0.27	7.29 ± 0.64
*h^2^*	0.52	0.82	0.49	0.6	0.72
Year 3*					
σ^2P^	8.40 ± 1.83	1.58 ± 0.29	7.88 ± 2.91	~	7.21 ± 1.42
σ^2P×S^	0.86 ± 0.97 ***	0.08 ± 0.08 ns	0.00 ns	~	0.00 ns
σ^2Er^	10.24 ± 1.13	1.12 ± 0.09	14.82 ± 2.17	~	6.56 ± 0.58
*h^2^*	0.64	0.82	0.49	~	0.76

Significance levels of interaction (*P< 0.05; *** P< 0.001).

**Table 6:** Estimated population (σ^2<sub>P</sub>^), population-by-season (σ^2<sub>P×S</sub>^), population-by-year (σ^2<sub>P×Y</sub>^), residual error (σ^2<sub>Er</sub>^); variance components, their associated interactions, and standard errors (± SE); and population mean narrow-sense heritability (*h^2^*) for plot height, plot density, and chlorophyll content for 92 red clover germplasm populations at Lincoln over 3 seasons.

Source of variation	Plot height	Plot Density	Chlorophyll content
Spring			
σ^2P^	7.80 ± 1.55	1.18 ± 0.24	3.40 ± 1.06
σ^2P×Y^	1.58 ± 0.73 ***	0.49 ± 0.11 ***	0.01 ± 0.86 ns
σ^2Er^	8.62 ± 0.76	1.33 ± 0.09	9.66 ± 1.02
*h^2^*	0.62	0.71	0.52
Autumn			
σ^2P^	4.36 ± 0.91	1.01 ± 0.23	4.26 ± 1.24
σ^2P×Y^	0.00 ns	0.14 ± 0.16 ***	0.33 ± 1.19 ns
σ^2Er^	7.57 ± 0.51	2.24 ± 0.20	16.74 ± 1.48
*h^2^*	0.66	0.67	0.47
Summer			
σ^2P^	3.51 ± 5.91	1.17 ± 0.29	4.82 ± 1.20
σ^2P×Y^	0.00 ns	0.13 ± 0.18 ***	0.00 ns
σ^2Er^	5.83 ± 2.21	1.81 ± 0.19	8.71 ± 0.75
*h^2^*	0.76	0.69	0.65

Significance levels of interaction (*** P< 0.001).

Narrow-sense heritability (*h^2^*) for biomass, growth habit, and leaf size was high (≥ 0.60) across most years, seasons, and locations, along with within years, seasons, and locations. Exceptions were at Palmerston North for biomass in year 3 (0.44) and year 1 spring (0.55), growth habit at Palmerston North for year 1 (0.26) and summer (0.26), along with growth habit across both sites in year 1 (0.55). At the Lincoln site, narrow-sense heritability was high (≥ 0.60) across and within years and seasons for plot height, plot density, lamina area and survival, except for plot height in year 2 (0.52) and survival in year 1 (0.09). By contrast, chlorophyll content narrow-sense heritability was high (≥ 0.60) only across years and summer.

### 3.5 Genetic diversity clustering and phenotypic trait expression showed commonality

**Figure 4:**
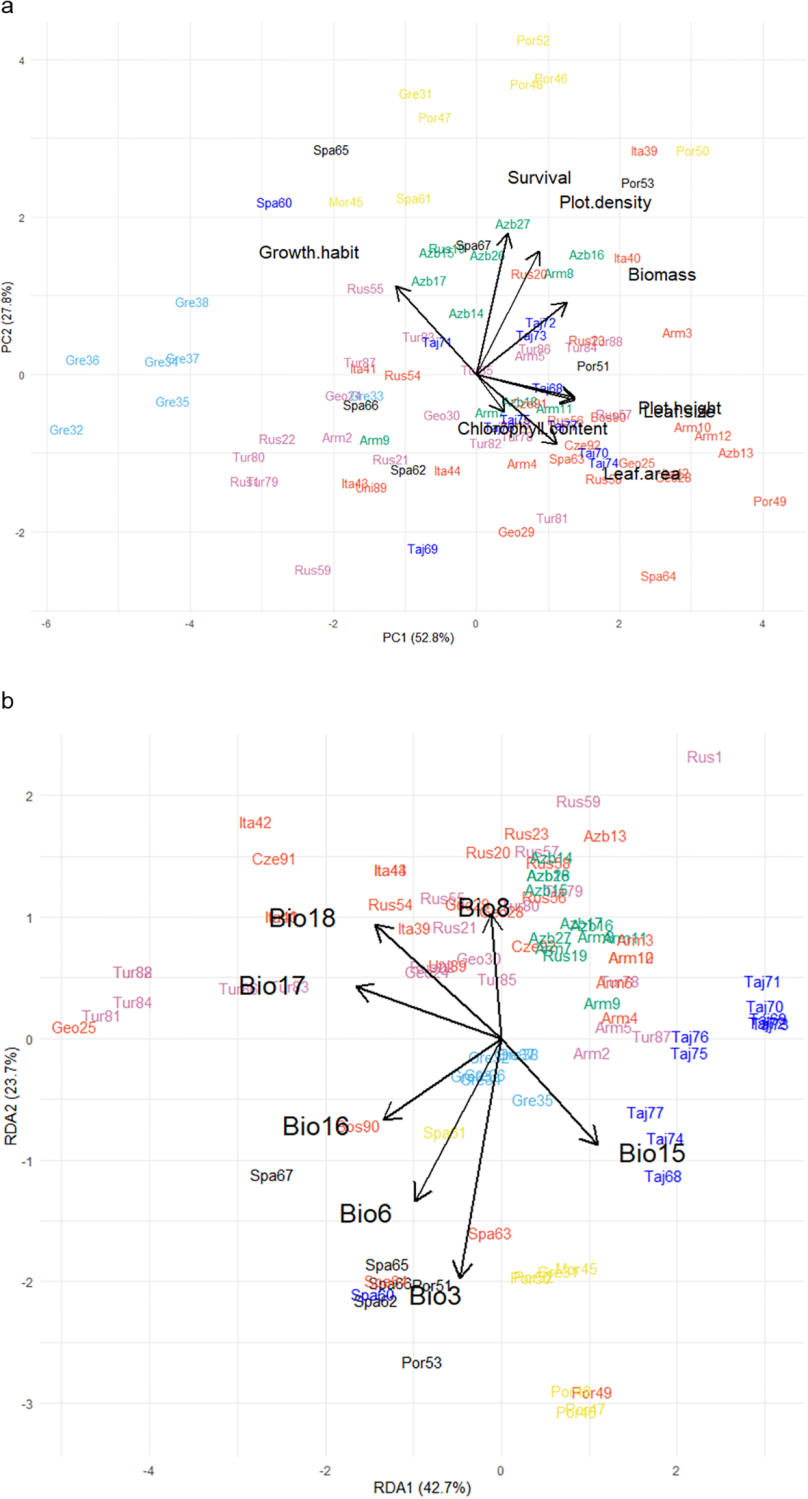
**(a)** Biplot generated using standardized Genomic Best Linear Unbiased Prediction values of 92 red clover germplasm populations assessed in a common garden experiment at Lincoln over 3 years for chlorophyll content, growth, growth habit, lamina area, leaf size, plot density, plot height, and survival. Populations were clustered into groups based on discriminant analysis of principal components (DAPC) analysis; groups are indicated by color and align with those in **Figure 1**. **(b)** Redundancy analysis biplot generated from pattern analysis using bioclimatic variables for the collection sites of the 92 germplasm populations. These were: Isothermality (Bio3); Min temperature of coldest month (Bio6); Mean temperature of driest quarter (Bio8); Precipitation seasonality (Bio15); Precipitation of wettest quarter (Bio16); Precipitation of driest quarter (Bio17); Precipitation of warmest quarter (Bio18). Populations were colored according to previous DAPC clustering described above.

Principal component analysis of the 92 populations’ performance, based on genomic best linear unbiased prediction values for a range of traits over three years at the Lincoln site, showed that the first two principal components explained 81.6% of the phenotypic variation among populations (**Figure 4a**). The first principal component (PC1) explained 52.8% of the variation and was driven primarily by plant biomass, leaf area, plot density, and plot height. The second principal component (PC2) explained 27.8% of variation and was underpinned by growth habit, survival, and plot density. Many of the traits had significant positive relationships with each other, particularly plot height and leaf size, and plot density and leaf area (**Figure 4a**). By contrast, growth habit, for which high scores indicated more prostrate plants, was strongly negatively correlated with chlorophyll content, and slightly less so with plot height and leaf size.

As described previously, the 92 populations were assigned to seven clusters based on genetic diversity analysis. Some member populations within these clusters also grouped similarly by distinctive phenotypic characteristics as expressed in a New Zealand field trial context. Cluster 7 (yellow), which comprised of eight populations predominantly from the Iberian Peninsula, Morocco, and southern Greece, was placed in near proximity on the biplot and had similar characteristics such as a semi-prostrate growth habit which produced high biomass and had high plot density and survival (**Figure 4a**). Likewise, the eight populations from Northern Greece (Cluster 5; light blue) formed a cluster and aligned according to phenotype and exhibited a short, small leaf, prostrate growth habit and produced low biomass. By contrast, Cluster 1 (Northern Spain; black), which contained six populations (**Figure 1**; **Supplementary Table 2**), each of which showed different phenotypes across the eight traits and were scattered around the biplot (**Figure 4a**).

The cosmopolitan Cluster 2 (red) contained 27 populations which, despite being sourced from many European or Caucasian countries (**Figure 1**; **Supplementary Table 2**), aligned phenotypically and were characterized by their erect, large leaf growth habit (**Figure 4a**). These populations produced high biomass but had lower plot density and survival. The 12 populations from the Eastern Caucasus (Cluster 3; green), were placed near the center of the biplot and exhibited average performance for most of the growth traits but some populations did have higher plot density and survival compared to most (**Figure 4a**). The neighboring Cluster 6 (purple) populations in western Caucasus and Eastern Turkey (**Figure 1**; **Supplementary Table 2**) were similar to Cluster 3 but had lower plot density and survival. Cluster 4 (dark blue) which contained 11 populations from Tajikistan and one from Spain were scattered near the center of the biplot and exhibited average growth, although some were categorized by their erect larger leaf growth habit and others with lower survival (**Figure 4a**).

### 3.6 Landscape genomics identified key environmental relationships

For the landscape genomics analysis, the best model was fitted based on the predicted influence of bioclimatic variables. Collinearity of these variables across the map locations of the source sites, which can skew the redundancy analysis, was accounted for through identification and removal of one in each pair of highly correlated variables (*r* ≥ 0.8). This resulted in seven out of the 19 bioclimatic variables (**Supplementary Table 1**) being retained for the analysis, and these were: Isothermality (Bio3); Minimum temperature of coldest month (Bio6); Mean temperature of driest quarter (Bio8); Precipitation seasonality (Bio15); Precipitation of wettest quarter (Bio16); Precipitation of driest quarter (Bio17); and Precipitation of warmest quarter (Bio18). The subsequent redundancy analysis evaluated patterns between allele frequency of the populations in response to these bioclimatic data based on the source map coordinates of the 92 populations. This showed that the first two principal components accounted for 66.4% of the allelic frequency variation where the first redundancy analysis component (RDA1) explained 42.7% of the allelic variation and was driven by precipitation bioclimatic variables (Bio15, 16, 17 and 18), and RDA2 (23.7% variation) was underpinned by temperature (Bio3, 6 and 8) (**Figure 4b**). Most of the environmental explanatory variables had positive relationships with each other, however Precipitation seasonality (Bio15) had a strongly inverse relationship with both Precipitation of warmest quarter (Bio18) and Precipitation of driest quarter (Bio17), and Mean temperature of driest quarter (Bio8) had a strong inverse relationship with Isothermality (Bio3) (**Figure 4b**).

The 92 populations on the RDA plot in response to climatic variables were clustered in groups that in many cases reflected the genetic diversity analysis (**Figure 4b**). Populations from discrete regions such as Northern Greece (Cluster 5; light blue) and Tajikistan (Cluster 4; dark blue) exhibited localized clustering on the RDA plot, whereas Cluster 6 (Turkey/Western Caucasus; pink) and the cosmopolitan Cluster 2 (red) from a range of environments were more widely dispersed (**Figure 4b**). The single Spanish-sourced population (Spa60, blue) that had been grouped with Cluster 4 (Tajikistan, blue), was placed with other Spanish material in the redundancy analysis (**Figure 4b**). The Tajikistan populations aligned strongly with seasonal precipitation (Bio15) whereas some Turkish (Tur, pink) and Italian (Ita, red) germplasm has strong relationships with drought and summer rainfall (Bio17 and 18). Material from the Caucasus region (Clusters 2 (red), 3 (green) and 6 (pink)) aligned with Mean temperature of the warmest quarter (Bio8) suggesting alleles associated with adaptation to summer heat. The distribution of populations from within genetic clusters across bioclimatic variables suggests that while there were sufficient alleles in common that underpinned genetic grouping, there were subsets associated with environmental adaptation that aligned populations from similar environments.

**Figure 5:**
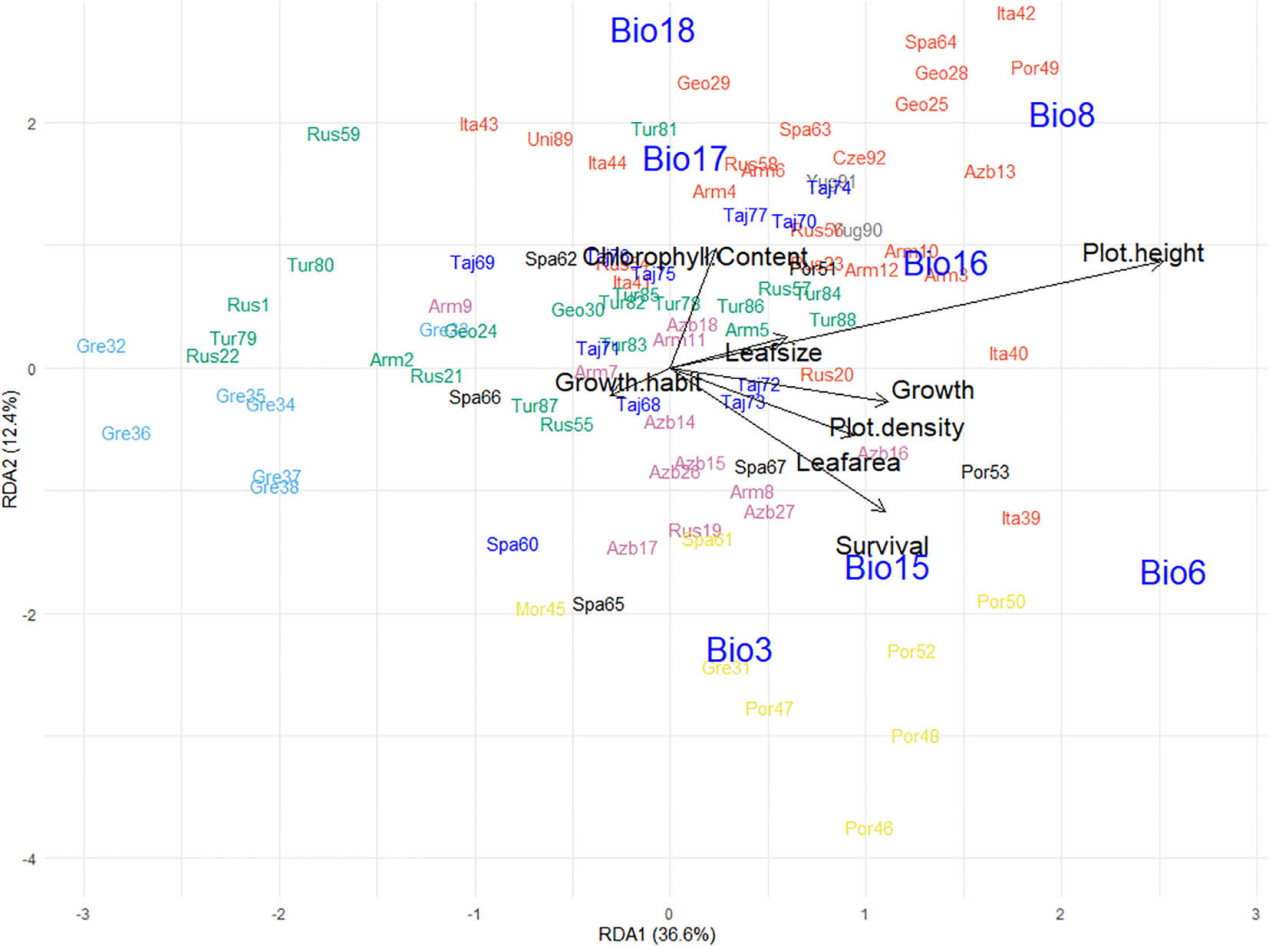
Redundancy analysis biplot generated from pattern analysis using standardized genomic best linear unbiased prediction values of 92 globally diverse red clover germplasm populations assessed at the common garden Lincoln site over 3 years for chlorophyll content, growth, growth habit, lamina area, leaf size, plot density, plot height, and survival. Combined with bioclimatic variables (Isothermality, Bio3; Min temperature of coldest month, Bio6; Mean temperature of driest quarter, Bio8; Precipitation seasonality, Bio15; Precipitation of wettest quarter, Bio16; Precipitation of driest quarter, Bio17; Precipitation of warmest quarter, Bio18) for the collection sites of the 92 germplasm populations. Populations were clustered to groups based on discriminant analysis of principal components (DAPC) analysis; groups are indicated by color aligned with **Figure 1**.

Incorporating data from a three-year trial at the Lincoln common garden site provided further insight into relationships between phenotype and underlying genetic drivers of local adaptation. This was derived from phenotype data aligned with genotype information and the source environmental explanatory factors (**Figure 5**). As when assessing allele frequency and bioclimatic variables alone, the 92 populations formed groups that aligned with their genetic clustering described previously. The addition of plant phenotype data (**Figure 5**) was found to tighten some of the clusters described in **Figure 4b**, along with identifying several notable relationships between trait response and environmental explanatory variables from where the populations came. An example of this was the cosmopolitan Cluster 2 (red), which formed a tight grouping compared to **Figure 4b**. This was influenced by chlorophyll content and aligned with Precipitation of Driest Quarter (Bio17) and Precipitation of Warmest Quarter (Bio18) (**Figure 5**). Precipitation of Wettest Quarter (Bio16) of the source material was strongly aligned with increased plot height and leaf size, which had an inverse relationship with the prostrate small-leaved tightly grouped Greek material in Cluster 5 (Light Blue). Populations from Portugal, Spain, Morocco and Southern Greece (Cluster 7, Yellow) performed best in the common garden which exhibited high growth, plot density, lamina area and plant survival and were strongly aligned with Minimum Temperature of Coldest Month (Bio6), Precipitation Seasonality (Bio15) and Isothermality (Bio3) of their origin.

### 3.7 Candidate SNPs associated with adaptation

**Figure 6:**
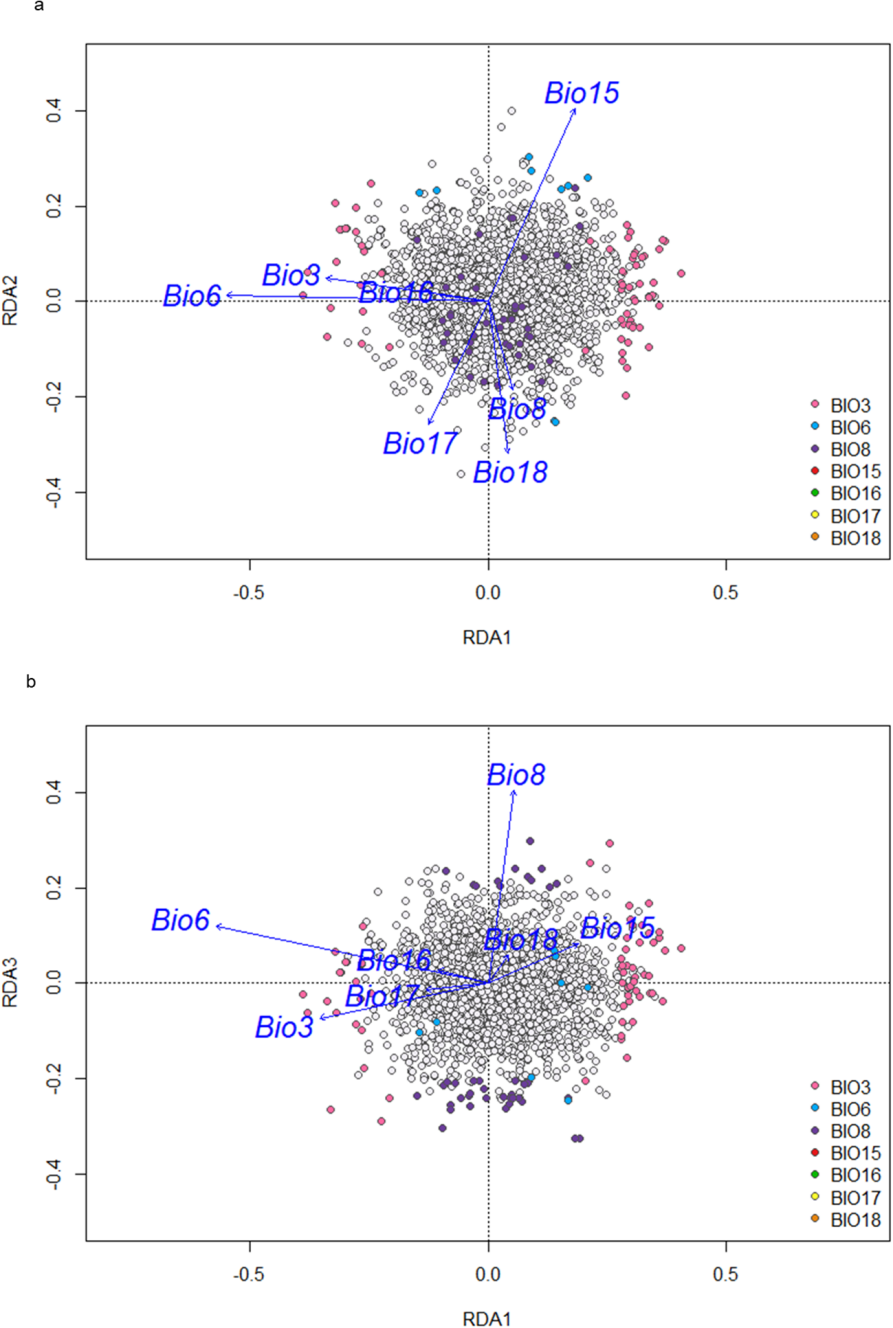
**(a)** All SNPs with candidate SNPs and their association with bioclimatic variables identified through redundancy analysis along RDA1 and RDA2. Candidate SNPs colored by the bioclimatic variable they are predominantly associated with. **(b)** All SNPs with candidate SNPs and their association with bioclimatic variables identified through redundancy analysis along RDA1 and RDA3. Candidate SNPs colored by bioclimatic variable they are predominantly associated with.

From the 4,509 SNPs used in the DAPC and subsequent analyses, redundancy analysis identified 123 SNPs as candidates associated with local adaptation based on population collection site eco-geographic data (**Figure 6**). These candidate SNPs were aligned with temperature bioclimatic variables as 69 SNPs were identified that were predominantly associated with Isothermality (Bio3), 9 SNPs with Minimum temperature of the coldest month (Bio6), and 44 SNPs with Mean temperature of driest quarter (Bio8). No SNPs were found to associate with precipitation variables (Bio15,16, 17 and 18). The location of each candidate SNP was based on the ARS RC 1.1 (GCA_020283565.1) red clover reference genome (Bickhart et al., 2022), and its position relative to a gene, either within, or no more than 3KB upstream or downstream was described (**Supplementary Table 3**). Based on these 123 SNPs, 89 separate genes were identified, of which 33 contained multiple SNPs (**Supplementary Table 3**). Most genes identified were protein coding (81) but pseudo genes (5), non-coding RNA (1), small nucleotide RNA (1) and one unknown were also identified. The majority of GO terms for the 89 genes associated with local adaptation were found to be at a high level and associated with metabolic process functions involved in photosynthesis, respiration, protein synthesis or nutrient absorption processes (**Supplementary Figure 4a**). For Isothermality (Bio3) and mean temperature of driest quarter (Bio8), GO terms were primarily associated with macromolecule metabolic and cellular processes (**Supplementary Figures 4b, c**).

## 4 Discussion

### 4.1 Population structure aligns with source regions

The center of origin for red clover is thought to be the Eastern Mediterranean region, from where it spread throughout Europe and western Asia (Annicchiarico et al., 2015; Taylor and Quesenberry, 1996). The 92 red clover populations were selected from across this range and were assigned to seven clusters based on their SNP allele frequencies. These clusters aligned with latitude from East to West Europe and reflect outcomes from previous red clover germplasm panels (Jones et al., 2020; Frey et al., 2022 and Zanotto et al., 2023). Identification of seven clusters based on Bayesian information criterion (BIC) to group the populations was corroborated as the seven clusters aligned predominantly with countries/regions. This coupled with the fact that the majority of variance being within populations showed a degree of local adaptation among populations from similar origins. In this study, describing the diversity of germplasm using a combined process of selecting a representative subset using eco-geographical factors then employing pooled-GBS to describe relationships among populations mostly showed strong alignment between ecogeographic clustering and the genetics. Notable exceptions were, however, Cluster 2 which was not associated with a specific region but spread across Europe and the Caucasus, and Cluster 7, which extended from Spain to Morocco and Southern Greece. This highlighted that in some cases, the genetic diversity information provided a more nuanced interpretation of population relatedness and insight into potential movement around a region than could be determined from using location data alone. However, the close alignment in majority of clusters validates that selection based on ecogeographic variables as a quicker resource conserving methodology. Furthermore, the use of bulk GBS in a population provided a cost-effective and efficient way to describe the genetic diversity. This technique has been used successfully for red clover, perennial ryegrass and white clover, all genetically diverse outcrossing species (Zanotto et al., 2021; Frey et al., 2022; Nay et al., 2023; Zanotto et al., 2023; Bryne et al., 2013 and Faville et al., 2020) and is a way to reduce genotyping costs significantly.

The highest biomass producing populations in our trials in New Zealand were from Cluster 7 and comprised populations that were spread throughout Spain, Portugal, Morrocco and Southern Greece. As cultivation of red clover has been reported in Spain from the 16^th^ century, it may be these populations are remnants of early cultivars or landraces spread by farmers (Annicchiarico et al., 2015; Taylor and Quesenberry, 1996), predisposing these populations to enhanced agronomic performance. Alternatively, the environmental parameters of the collection and evaluation sites in particular isothermality, temperature of the coldest month and precipitation seasonality shared similarities which could have favored the performance of these populations in the New Zealand environment. This is supported further through the development and release in recent decades of high performing cultivars ‘Grasslands Broadway’, ‘Grasslands Crossway’, ‘Grasslands Colenso’ and ‘Grasslands Relish’ which contain either Spanish, Moroccan or Portuguese genetic backgrounds (Rumball et al., 2003; Claydon et al., 1993; Ford and Barrett, 2011). This suggests populations from these regions have genetics suitable for performance in New Zealand.

Based on the data in this study, it appears that the Caucasus Mountain range is a geographic hotspot for variation amongst the populations in this study. Of the seven groups, two were located here and the cosmopolitan Cluster 2 overlapped with this region in addition to Southern/Central Europe and the Iberian Peninsula. The Caucasus Mountain range are known for highly variable and localized landforms, climates and seasonal characteristics which are difficult to summarize for large areas (Greene et al., 1999). Previous studies in both red and white clover have identified this region as a center of diversity for heterogeneous populations mainly due to topographic separation and isolation (Greene et al., 1999, 2004; Mosjidis et al., 2004; Caradus and Forde, 1996; Faville et al., 2020). This region is a potentially rich source of genetic diversity.

Another measure of genetic diversity is the expected heterozygosity which indicates the proportion of different alleles in the populations. While our data suggested that there was high gene flow mediated by pollen or seeds among the seven clusters, the variation in expected heterozygosity for our populations was at the lower end of levels found in other studies (Mosjidis and Klinger, 2006; Jones et al., 2020; Osterman et al., 2021). This reduced expected heterozygosity could be attributed to the restricted sample size (30 individuals) we used to develop the bulk DNA samples, although previous work noted that within-population genetic diversity of out-crossing populations can be determined using at least 20 individuals per population (Khanlou et al., 2011). It may also reflect that the earlier investigations had fewer individuals per population sampled which could have had an upward pressure on expected heterozygosity as, for example, previous studies with higher expected heterozygosity had between 8 and 18 individuals per population (Mosjidis and Klinger, 2006; Jones et al., 2020; Osterman et al., 2021). Additionally, red clover is well known for its high genetic diversity, and partitioning the genetic variance using AMOVA in this study identified higher levels of variation was found among-population (79%) compared to among (14.6%). This is expected in outcrossing species and reflects previous studies on this species (Kölliker et al., 2003; Pagnotta et al., 2011; Dias et al., 2008). This abundance of diversity provides an advantage for breeding programs but does cause issues with identifying and generating an accurate representation of this diversity to be conserved in a genebank (Dias, et al., 2008; Osterman et al., 2021; Taylor and Quesenberry, 1996).

### 4.2 Limitation of a pedigree-based approach to assessing and evaluating germplasm

When assessing collections of raw germplasm, the efficiency and accuracy of a pedigree-based approach will be limited depending on the amount of information available (Egan et al., 2020). Due to the smaller number of populations combined with a high number of founder populations present, there were limitations in using a pedigree-based mapping tool to describe relationships amongst our populations. A pedigree relationship or A-matrix could not be generated for incorporation into the linear mixed model, but we could confirm a high level of diversity amongst our populations as none of the 77 populations with some pedigree information available showed any relatedness or inbreeding. A genomic relationship matrix based on the DNA, therefore, provides a more accurate representation of population structure which reflects the genetic pedigree of the samples at hand, whereas a standard pedigree-based approach requires accurate data recording across a number of generations. The usefulness of pedigree information is not in question, due to a well-recorded benefit of being a low-cost tool to improve accuracy when integrated with genomic prediction models, but is better suited for crops such as wheat which have well established multi-generational pedigree lines which many forage species lack (Cericola et al., 2018; Crossa et al., 2010; Egan et al., 2020, 2019).

### 4.3 Significant narrow-sense heritability and genetic variance identified

Overall, the high narrow-sense heritability (*h^2^* > 0.60) for most of the eight traits (biomass, growth habit, leaf size, plant height, plant density, chlorophyll content, leaf area and survival) suggests that improvement of these traits can be attained via breeding. When scrutinizing the data at a site level, however, growth habit and leaf size additive/population variance components, hence narrow-sense heritability values, were not significant at the Palmerston North site across years and seasons. This may be due to fewer seasonal measurements being recorded, two for each trait compared to nine at the Lincoln site due to limited resources. There were fewer measurements at the Palmerston North site due to issues with controlling volunteer white clover amongst plots. Fewer measurements can make it difficult to distinguish the proportion of trait variance due to genetic versus environmental effect.

For both physiological and morphological traits there was, however, large genetic variation for trait expression, a trend also found in other red clover germplasm trials (Annicchiarico and Pagnotta, 2012; Dias et al., 2008; Greene et al., 2004; Kouamé and Quesenberry, 1993; Pagnotta et al., 2011; Zanotto et al., 2021; Nay et al., 2023). Morphological variation has been shown to strongly interact with environmental variation through the expression of different phenotypes in different environments (Greene et al., 2004). As this is unimproved germplasm material, most observed variation in plant type or morphological traits is a direct reflection of natural selective pressures from original collection sites (Annicchiarico and Pagnotta, 2012). The large amount of variation among our populations reflects the diverse collection regions and local habitats. Additional exploration of the variation within these populations is important, especially in additional diverse environments. This would add to the precision and utilization of predictive models based on genomic, phenotypic, and environmental information. However, the high heritability and diversity observed for these traits indicate the potential for trait improvement through the selected addition of populations into existing breeding pools.

### 4.4 Local adaptation and phenotypic trait expression characterized among populations

When assessing the phenotypic diversity among the seven clusters, the amount of trait variation was extensive. A noted pattern was the commonality of the phenotype of populations placed within the same clusters based on genotype data. This suggests that the commonality of environment has shaped the genetics and the phenotype, indicating populations in a region have genetics and phenotypic traits in common. When focusing on specific phenotypes, the populations in Cluster 5 (Northern Greece) are of interest due to their very prostrate growth habit. These populations more closely resembled a white clover creeping plant type compared to a typical erect red clover growth habit. Similar growth habit has been observed in the red clover cultivars ‘Astred’, ‘Grasslands Crossway’, ‘Grasslands Broadway’ and ‘Rubitas’, which are known for their prostrate stoloniferous growth habit (Hyslop et al., 1998; Rumball et al., 2003; Watson et al., 2015). These attributes enable them to better withstand repeated defoliation, which underpins persistence as a grazed red clover. Although, as observed in this grazed trial and previous work, this growth habit is compromised as biomass is severely reduced compared to more erect populations (Jones et al., 2020; Riday, 2009; Mirzaie-Nodoushan et al., 1999; Zanotto et al., 2021). However, although these populations themselves may not produce large amounts of biomass, they can be crossed with more erect high biomass producing types to increase the persistency and performance of the semi-prostrate/semi-erect progeny in a mixed sward grazing system (Mirzaie-Nodoushan et al., 1999).

The persistency of semi-erect populations is shown by the populations in Cluster 7 (Iberian Peninsula, Morocco, Southern Greece). These populations were found to produce high biomass and a higher survival rate in the field trials and tended to possess a medium-sized leaf and plot height. These populations would make a great addition to or a starting point for a breeding program for these environments and could be enhanced by incorporating more prostrate material from Cluster 5 (described above) as a route to increasing persistence further. The positive associations between erect growth habit, plot height, and leaf size, a feature of Cluster 7 populations, have also been found in other red clover studies (Annicchiarico and Pagnotta, 2012; Pagnotta et al., 2011; Mauro et al., 2011; Zanotto et al., 2021). Similarly, the negative association between growth habit and chlorophyll observed has also been reported in both red and subterranean (*Trifolium subterranean* L.) clovers (Brougham, 1960; Mauro et al., 2011). This is attributed to the prostrate plants being more prone to shading from companion species such as ryegrass which reduces the number of leaves and stalk exposed, minimizing the potential amount of light that can be intercepted. In turn, this reduces the efficiency of the photosynthetic process, limiting the amount of energy produced (Brougham, 1960; Mauro et al., 2011).

### 4.5 Key interactions between performance, trait response and environment identified

Landscape genomics is an interdisciplinary methodology that investigates the influence of environmental variables on the distribution of genetic variation within and amongst populations (Bragg et al., 2015). It is, however, an under-utilized tool with few publications in crop species, and none to date in red clover. The most recent studies have focused on adaptation to climatic variables in forest species such as lodgepole pine (*Pinus contorta*), red spruce (*Picea rubens Sarg*.) and common beech (*Fagus sylvatica*) (Capblancq and Forester, 2021; Capblancq et al., 2023, 2020). Other studies have focused on barley (*Hordeum vulgare* L.), Arabidopsis (*Arabidopsis thaliana L*.), and poplar tree species (*Populus trichocarpa* L. and *Populus balsamifera* L.) (Abebe et al., 2015; Lasky et al., 2014; Evans et al., 2014; Fitzpatrick and Keller, 2015). The influence of soil environments on adaptation for barrel medic (*Medicago truncatula* L.) has also been investigated (Guerrero et al., 2018). These studies showed how landscape genomics can contribute to identifying genetic variation underpinning stress response and local adaptation and how this information may be incorporated into predictive models to assess the impact of environmental change.

Redundancy analysis allowed us to identify key associations between population performance, trait response and environmental explanatory factors using the common garden Lincoln site. A notable association was between chlorophyll content, used here as a proxy for photosynthetic capacity, and the level of precipitation of the driest and warmest quarters in the geographic sites from which the populations were sourced. Both precipitation and temperature play an important role in the photosynthetic capacity of plants (Taylor and Quesenberry, 1996) and identifying populations capable of maintaining high levels of photosynthesis during periods of stress is important for the introduction of key adaptive traits into future cultivars.

Precipitation seasonality (Bio15), the irregular distribution of rain over a year, was identified as a key environmental factor that influenced the growth traits including chlorophyll content, biomass, plot density, lamina area and survival. The timing of rainfall events is crucial for plant growth, especially leading into and exiting periods of stress such as drought or cold, where plants look to replenish or increase stored carbohydrates used to promote growth (Taylor and Quesenberry, 1996). The performance of populations in Clusters 3 (eastern Caucasus), 4 (Tajikistan) and 7 (Iberia/Morocco) in the field trials on the drought-prone east coast of New Zealand (Lincoln) had the strongest association with precipitation seasonality of their source locations. These three groups originate from either mountainous or steep coastal areas, which have irregular rainfall. It may be that this precipitation irregularity in the source regions primed the populations for suitability to New Zealand environments as evidenced by the success of these populations in our field trials.

Isothermality (Bio3) refers to the level of variance in temperature throughout the year and has strong relationships with plant survival and growth habit (Osterman et al., 2021). The change between seasons can be critical for plant survival especially entering and exiting periods of stress such as drought or cold. A plant must be able to replenish stored carbohydrates key for the promotion of growth over these periods of stress (Black et al., 2009). It is expected that having less variance between temperature extremes throughout the year would allow the plants to be less stressed without having to adapt to large temperature fluctuations. The performance of Clusters 1 and 7, both predominantly of Spanish and Portuguese origins had the strongest association with isothermality in terms of their performance at the common garden Lincoln site. The mediterranean climate of both Spain and Portugal means they have hot dry summers and very mild winters which aligns with the New Zealand sites. This suggests that material from this region is primed to perform well as shown by the success in our field trials. However, other factors such as grazing pressure, pest, diseases, and soil nutrition will impact how well these populations will adapt and perform.

### 4.6 Candidate SNPs associated with adaptation

The identification of the 123 candidate SNPs that were predominantly associated with bioclimatic variables provide a route to identifying candidate genes and genetic regions that could influence phenotypic adaptation to local conditions (Bragg et al., 2015). While the majority of the identified genes had a protein coding function which could be associated with key cell functions, further investigation into the role each of these genes has on plant performance is needed. However, a set of 57 of our candidate SNPs and associated genes aligned with a previous study assessing drought response and tolerance in red clover through differential gene expression amongst droughted and non-droughted red clover plants (Yates et al., 2014). The 57 genes in common with this drought trial were linked to temperature variables with 32 solely associated with Isothermality (Bio3), 21 with Mean temperature of driest quarter (Bio8), two with Minimum temperature of coldest month (Bio6) and one to all three bioclimatic variables in our study. Of these 57 SNPs, three and their associated genes were also identified by Yates et al. (2014) as being present in drought tolerant plants (**Supplementary Table 4**). These genes could be a potential SNP marker linked to drought tolerance.

Investigating the Gene Ontology (GO) terms of the 123 SNPs/genes associated with the bioclimatic variables variable to gain some insight as to the functionality of these genes was not conclusive. The majority of GO terms were found to be at a high level, which may be due to there being several variables and stress responses influencing gene expression and for each environment. It may reflect the annotation of the genome or that only 123 genes were assessed compared with the multiple thousands of differentially expressed genes in a typical transcriptome analysis. For example, exposure of white clover to frost revealed ~3,200 upregulated transcripts, and GO analysis of these showed greater enrichment for and resolution of the pathways involved, particularly the expected plant stress responses (Fechete et al., 2024), compared to the 123 genes we investigated. Even so, the commonality of some SNP-associated genes with previous red clover drought work, and the association of a set of SNP-associated gene with bioclimatic variables data strengthens the need to further investigate the role and influence of these genetic regions and how they may be used in selection for trait enhancement.

### 4.6 Conclusions and future steps

This study assessed the genetic diversity and genetic relationships among 92 red clover raw germplasm populations tested in environments different to where they are adapted and highlighted an abundance of potential diversity available in untapped germplasm material. We applied a range of methodologies including common garden trials, genetic relationship models, pedigree relationship matrix and landscape genomics to assess this material. All methodologies provided different insights and usefulness. The limitations of the pedigree-based mapping tools were highlighted, with genomic relationship matrices better reflecting relatedness amongst populations with little pedigree information, a feature of many forage crops, especially where there are many populations recognized as being founders. The benefit of conducting multi-year, multi-location, multi-grazing pressure field trials was emphasized through quantifying the magnitude of genotype and G × E variances for key agronomic and physiological traits. Key interactions between plant performance and survival with these traits were also identified. The influence of environmental variables from original collection locations on local adaptation through plant and trait performance and the identification of candidate alleles was highlighted. The alignment between the genetic diversity and the ecogeographic information showed the diverse nature of the selected plant material. It also gave confidence in using quality ecogeographic information for selecting populations from genebanks, but also highlighted the advantage of using genotype data for the genetic insight which is independent and may be used to corroborate genebank labeling/passport information. As the populations are genetically separate and diverse it enables us to focus on using landscape genomics to identify markers associated with adaptation to those ecogeographic regions, with the potential to harness these in future breeding decisions.

Further investigation is needed to determine the accuracy and importance of these methods for selecting germplasm material to introduce into breeding programs. The approach in this study was based on the ecogeographic information followed by a molecular marker characterization to assess diversity. A future approach to capture more diversity before initial field trialing would be to identify a larger cohort of accessions then perform the genetic diversity analysis to select a smaller number of accessions based on maximizing ecogeographical and genetic diversity although this will require more resources. However, data from the current study showed that material can perform well agronomically when placed into similar environments such as with the populations in Cluster 7 (Por/Spa/Mor/Gre), particularly when assessed in New Zealand’s dry east coast. It also highlighted that raw populations from other areas do not necessarily perform agronomically well in those same new locations. This emphasizes that the next step to access the novel genetics available in this material should be to cross these individuals to locally-adapted material. Although resource intense, it is an important and logical progression from testing raw germplasm populations to assessing how these new genetics perform when incorporated into populations adapted for local environments. This is where the impact of adaptive markers identified by landscape genomics can be validated in testing of this material. Therefore, plans are in place to follow up this study with the selection of locally adaptive elite material and top performing germplasm populations to cross and subsequently field test.

## Data Availability

The datasets presented in this study can be found in online repositories. The names of the repository/repositories and accession number(s) can be found in the article/**Supplementary Material**.
