# Supplementary material for: Local adaptation, genetic diversity and key environmental interactions in a collection of novel red clover germplasm
Source: Front Plant Sci. 2025 Mar 17;16:1553094. doi: 10.3389/fpls.2025.1553094 (PMC11955710; doi:10.3389/fpls.2025.1553094)
Supplement: Supplementary file 1 [file DataSheet1.docx]

Supplementary Material

Supplementary Table 1: The 19 bioclimatic variables sourced from the ‘WorldClim’ database (*worldclim.org*).

| **Variable** | **Units** | **Interpretation** |
| --- | --- | --- |
| Annual Mean Temperature (Bio1) | °C | The mean temperature for the year. |
| Annual Mean Diurnal Range (Bio2) | °C | The mean of monthly temperatures which shows temperature fluctuations. |
| Isothermality (Bio3) | % | Derived by calculating the ratio of Bio2 to Bio7 and then multiplying by 100. |
| Temperature Seasonality (Bio4) | % | The amount of temperature variation over a given period based on the ratio of the standard deviation of the monthly mean temperatures to the mean monthly temperature. |
| Max Temperature of Warmest Month (Bio5) | °C | The maximum monthly temperature occurrence over a given year. |
| Min Temperature of Coldest Month (Bio6) | °C | The minimum monthly temperature occurrence over a given year. |
| Annual Temperature Range (Bio7) | °C | A measure of temperature variation over a given period. |
| Mean Temperature of Wettest Quarter (Bio8) | °C | The mean temperatures during the wettest season. |
| Mean Temperature of Driest Quarter (Bio9) | °C | The mean temperatures during the driest season. |
| Mean Temperature of Warmest Quarter (Bio10) | °C | The mean temperatures during the warmest season. |
| Mean Temperature of Coldest Quarter (Bio11) | °C | The mean temperatures during the coldest season. |
| Annual Precipitation (Bio12) | mm | The sum of all total monthly precipitation values. |
| Precipitation of Wettest Month (Bio13) | mm | The total precipitation during the wettest month. |
| Precipitation of Driest Month (Bio14) | mm | The total precipitation during the driest month. |
| Precipitation Seasonality (Bio15) | % | The measure of the variation in monthly precipitation totals over the course of the year |
| Precipitation of Wettest Quarter (Bio16) | mm | The mean precipitation during the wettest season. |
| Precipitation of Driest Quarter (Bio17) | mm | The mean precipitation during the driest season. |
| Precipitation of Warmest Quarter (Bio18) | mm | The mean precipitation during the warmest season. |
| Precipitation of Coldest Quarter (Bio19) | mm | The mean precipitation during the coldest season. |

Supplementary Table 2: Original accession numbers and country of origin information for each population from the Margot Forde Genebank (MFG).

| **Line number** | **Accession Number** | **Origin** | **Line** | **Line number** | **Accession Number** | **Origin** | **Line** |
| --- | --- | --- | --- | --- | --- | --- | --- |
| 1 | 4125 | Russia | Rus1 | 47 | 3514 | Portugal | Por47 |
| 2 | 3887 | Armenia | Arm2 | 48 | 3512 | Portugal | Por48 |
| 3 | 3861 | Armenia | Arm3 | 49 | 3511 | Portugal | Por49 |
| 4 | 3860 | Armenia | Arm4 | 50 | 3061 | Portugal | Por50 |
| 5 | 3885 | Armenia | Arm5 | 51 | 3592 | Portugal | Por51 |
| 6 | 3717 | Armenia | Arm6 | 52 | 2496 | Portugal | Por52 |
| 7 | 3857 | Armenia | Arm7 | 53 | 2498 | Portugal | Por53 |
| 8 | 3898 | Armenia | Arm8 | 54 | 4100 | Russia | Rus54 |
| 9 | 3895 | Armenia | Arm9 | 55 | 4098 | Russia | Rus55 |
| 10 | 3702 | Armenia | Arm10 | 56 | 4092 | Russia | Rus56 |
| 11 | 3862 | Armenia | Arm11 | 57 | 4089 | Russia | Rus57 |
| 12 | 3891 | Armenia | Arm12 | 58 | 4094 | Russia | Rus58 |
| 13 | 3473 | Azerbaijan | Azb13 | 59 | 4090 | Russia | Rus59 |
| 14 | 3474 | Azerbaijan | Azb14 | 60 | 3595 | Spain | Spa60 |
| 15 | 3476 | Azerbaijan | Azb15 | 61 | 3590 | Spain | Spa61 |
| 16 | 3470 | Azerbaijan | Azb16 | 62 | 2647 | Spain | Spa62 |
| 17 | 3271 | Azerbaijan | Azb17 | 63 | 3071 | Spain | Spa63 |
| 18 | 3275 | Azerbaijan | Azb18 | 64 | 3594 | Spain | Spa64 |
| 19 | 2461 | Russia | Rus19 | 65 | 2538 | Spain | Spa65 |
| 20 | 4095 | Russia | Rus20 | 66 | 2507 | Spain | Spa66 |
| 21 | 4096 | Russia | Rus21 | 67 | 3591 | Spain | Spa67 |
| 22 | 4097 | Russia | Rus22 | 68 | 3946 | Tajikistan | Taj68 |
| 23 | 4088 | Russia | Rus23 | 69 | 3935 | Tajikistan | Taj69 |
| 24 | 2465 | Georgia | Geo24 | 70 | 3937 | Tajikistan | Taj70 |
| 25 | 3276 | Georgia | Geo25 | 71 | 3941 | Tajikistan | Taj71 |
| 26 | 3269 | Azerbaijan | Azb26 | 72 | 3948 | Tajikistan | Taj72 |
| 27 | 3270 | Azerbaijan | Azb27 | 73 | 3949 | Tajikistan | Taj73 |
| 28 | 3882 | Georgia | Geo28 | 74 | 3945 | Tajikistan | Taj74 |
| 29 | 3858 | Georgia | Geo29 | 75 | 3943 | Tajikistan | Taj75 |
| 30 | 3273 | Georgia | Geo30 | 76 | 3947 | Tajikistan | Taj76 |
| 31 | 4023 | Greece | Gre31 | 77 | 3942 | Tajikistan | Taj77 |
| 32 | 4079 | Greece | Gre32 | 78 | 3971 | Turkey | Tur78 |
| 33 | 4084 | Greece | Gre33 | 79 | 3332 | Turkey | Tur79 |
| 34 | 4085 | Greece | Gre34 | 80 | 3337 | Turkey | Tur80 |
| 35 | 4019 | Greece | Gre35 | 81 | 3331 | Turkey | Tur81 |
| 36 | 4077 | Greece | Gre36 | 82 | 3329 | Turkey | Tur82 |
| 37 | 4087 | Greece | Gre37 | 83 | 3335 | Turkey | Tur83 |
| 38 | 4080 | Greece | Gre38 | 84 | 3320 | Turkey | Tur84 |
| 39 | 3069 | Italy | Ita39 | 85 | 3347 | Turkey | Tur85 |
| 40 | 3060 | Italy | Ita40 | 86 | 3346 | Turkey | Tur86 |
| 41 | 3067 | Italy | Ita41 | 87 | 3349 | Turkey | Tur87 |
| 42 | 3070 | Italy | Ita42 | 88 | 3339 | Turkey | Tur88 |
| 43 | 3059 | Italy | Ita43 | 89 | 3057 | United Kingdom | Uni89 |
| 44 | 3068 | Italy | Ita44 | 90 | 3278 | Yugoslavia (Bosnia and Herzegovina) | Yug90 |
| 45 | 3532 | Morocco | Mor45 | 91 | 3277 | Yugoslavia (Croatia) | Yug91 |
| 46 | 3513 | Portugal | Por46 | 92 | 3102 | Czech Republic | Cze92 |


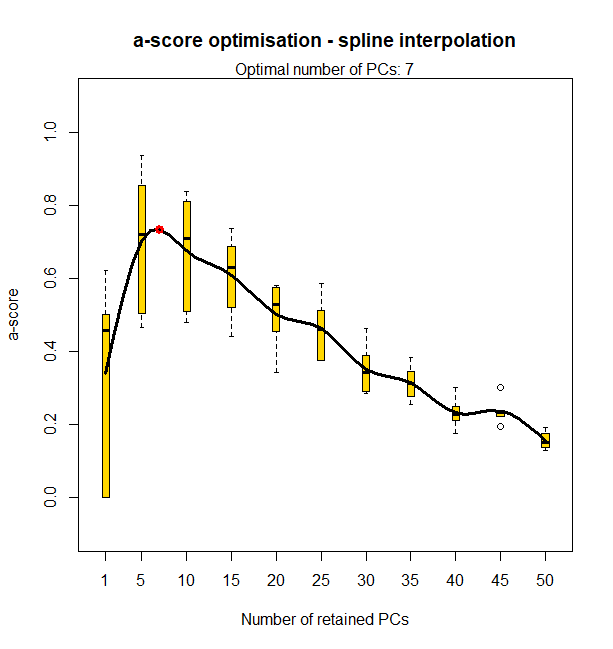


Supplementary Figure 1: A-score figure identifying the optimal number of principal components (7) to be used in the discriminant analysis of principal components (DAPC) analysis.


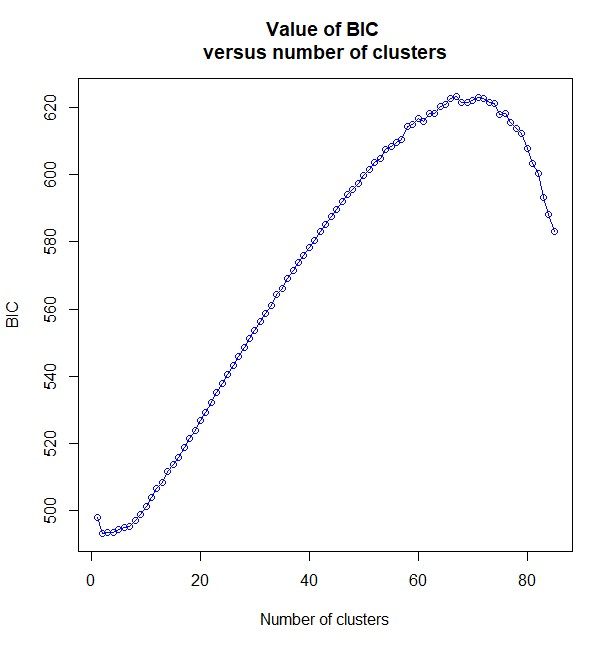


Supplementary Figure 2: Output from the FindCluster (K-mer clustering) as a plot of the Bayesian information criterion (BIC) values against cluster number. Where the BIC values are lowest indicates the optimal number of clusters among the populations tested.


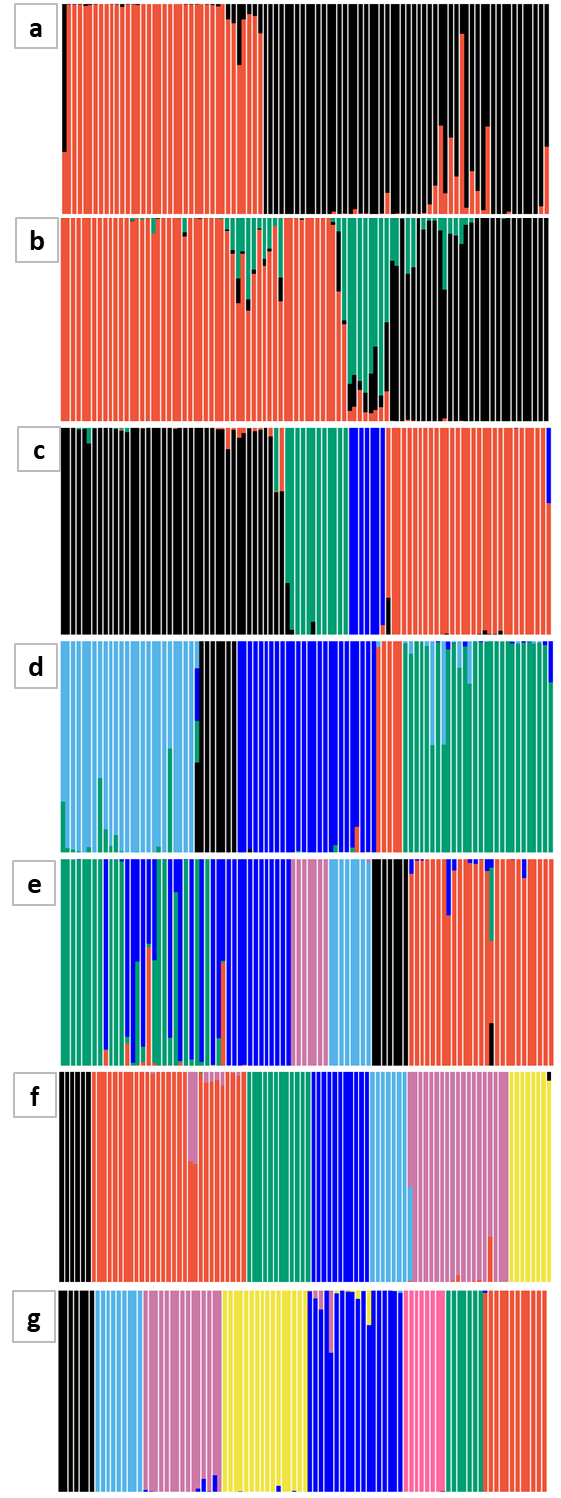


Supplementary Figure 3; Membership probability graphs for all 92 populations based on 2 to 8 clusters for a to g, respectively.

Supplementary Table 3; The structure of each cluster. Populations identified by country and population number Armenia (Arm), Azerbaijan (Azb), Bosnia & Herzegovina (Bos), Croatia (Cro), Czech Republic (Cze), Georgia (Geo), Greece (Gre), Italy (Ita), Morocco (Mor), Portugal (Por), Russia (Rus), Spain (Spa), Tajikistan (Taj), Turkey (Tur), and United Kingdom (Uni).

| Cluster 1 | Cluster 2 | | Cluster 3 | | Cluster 4 | | Cluster 5 | | Cluster 6 | | Cluster 7 | |
| --- | --- | --- | --- | --- | --- | --- | --- | --- | --- | --- | --- | --- |
| Por51 | Arm10 | Arm11 | | Spa60 | | Gre32 | | Arm2 | | Gre31 | |  |
| Por53 | Arm12 | Arm7 | | Taj68 | | Gre33 | | Geo24 | | Mor45 | |  |
| Spa62 | Arm3 | Arm8 | | Taj69 | | Gre34 | | Geo30 | | Por46 | |  |
| Spa65 | Arm4 | Arm9 | | Taj70 | | Gre35 | | Rus1 | | Por47 | |  |
| Spa66 | Arm5 | Azb14 | | Taj71 | | Gre36 | | Rus21 | | Por48 | |  |
| Spa67 | Arm6 | Azb15 | | Taj72 | | Gre37 | | Rus22 | | Por50 | |  |
|  | Azb13 | Azb16 | | Taj73 | | Gre38 | | Rus55 | | Por52 | |  |
|  | Bos90 | Azb17 | | Taj74 | |  | | Rus57 | | Spa61 | |  |
|  | Cze91 | Azb18 | | Taj75 | |  | | Rus59 | |  | |  |
|  | Cze92 | Azb26 | | Taj76 | |  | | Tur78 | |  | |  |
|  | Geo25 | Azb27 | | Taj77 | |  | | Tur79 | |  | |  |
|  | Geo28 | Rus19 | |  | |  | | Tur80 | |  | |  |
|  | Geo29 |  | |  | |  | | Tur81 | |  | |  |
|  | Ita39 |  | |  | |  | | Tur82 | |  | |  |
|  | Ita40 |  | |  | |  | | Tur83 | |  | |  |
|  | Ita41 |  | |  | |  | | Tur85 | |  | |  |
|  | Ita42 |  | |  | |  | | Tur86 | |  | |  |
|  | Ita43 |  | |  | |  | | Tur87 | |  | |  |
|  | Ita44 |  | |  | |  | | Tur88 | |  | |  |
|  | Por49 |  | |  | |  | |  | |  | |  |
|  | Rus20 |  | |  | |  | |  | |  | |  |
|  | Rus23 |  | |  | |  | |  | |  | |  |
|  | Rus54 |  | |  | |  | |  | |  | |  |
|  | Rus56 |  | |  | |  | |  | |  | |  |
|  | Rus58 |  | |  | |  | |  | |  | |  |
|  | Spa63 |  | |  | |  | |  | |  | |  |
|  | Spa64 |  | |  | |  | |  | |  | |  |
|  | Tur84 |  | |  | |  | |  | |  | |  |
|  | Uni89 |  | |  | |  | |  | |  | |  |

Supplementary Table 3; Bio climatic variables and the predominantly associated SNP information including chromosome number, SNP position and location relative to identified associated allele. Correlations and p-values for the association between SNPs and alleles. Significance levels of associations (*P< 0.05; **P< 0.01; *** P< 0.001). Allele information includes type, description and referenced literature.


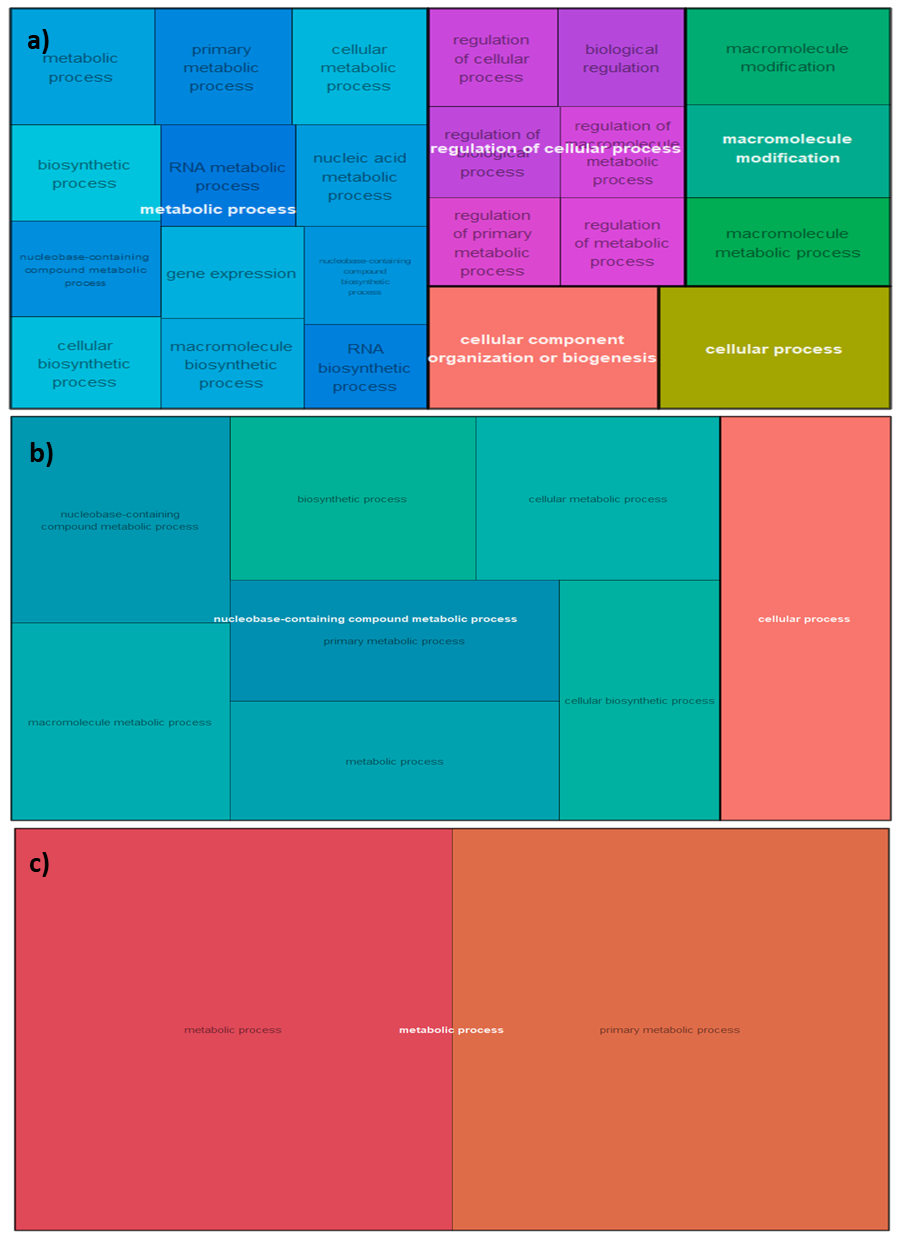


Supplementary Figure 4; Treemap view of the GO terms enriched in a) All Bios (Bios3, Isothermality; Bios6, Min temperature of coldest month; Bios8, Mean temperature of driest quarter; Bios15, Precipitation seasonality; Bios16, Precipitation of wettest quarter; Bios17, Precipitation of driest quarter; Bios18, Precipitation of warmest quarter); b) Bios3 (Isothermality); c) Bios8 (Mean temperature of driest quarter). Bios6, Bios15, Bios16, Bios17 and Bios18 didn’t have the necessary number of genes and therefore associated GO terms to generate individual Treemaps.
